# Temporal Dynamics of Groundwater Levels: A Complex Networks-Based Approach

**DOI:** 10.3390/e28101068

**Published:** 2026-09-28

**Authors:** Bhadran Deepthi, Bellie Sivakumar

**Affiliations:** 1 Kerala State Council for Science, Technology and Environment, Pattom, Thiruvananthapuram 695004, India; deepthibhadran2@gmail.com; 2 Department of Civil Engineering, Indian Institute of Technology Bombay, Powai, Mumbai 400076, India

**Keywords:** groundwater levels, complexity, chaos theory, complex networks, United States

## Abstract

This study explores the application of complex network theory to examine the temporal dynamics of groundwater levels in a region. It presents the first ever application of a coupled chaos theory–complex network framework to investigate the temporal dynamics and connectivity in groundwater levels in a region. For implementation, daily groundwater-level data observed over the period 2010–2014 from each of 71 wells across the United States are considered. Each well is considered as a network. In order to construct the temporal network from groundwater time series, basic concepts of chaos theory are coupled with those of complex network theory. First, the phase-space reconstruction technique is used to reconstruct the single-variable groundwater time series in a multi-dimensional phase space. Next, the optimum dimension required for the phase-space reconstruction is determined using the false nearest neighbour (FNN) algorithm. Each reconstructed vector in the phase space is considered as a node in the network, and the connections between the nodes (i.e., links) are identified based on the distance threshold between the reconstructed vectors. Four complex networks-based measures—degree centrality, betweenness centrality, closeness centrality, and clustering coefficient—are used to examine the properties of the groundwater-level network. The optimal embedding dimensions from the FNN method for the 71 groundwater level time series are found to range from 4 to 18, suggesting a wide range of complexity in the 71 groundwater level time series. However, a large majority (i.e., 62) of the time series have dimensions in the range of 4–10, suggesting low-to-medium-level complexity of the groundwater level dynamics. The results for the four network measures are degree centrality in the range 0.12–0.55, betweenness centrality in the range 604–4750, closeness centrality in the range 0.00009–0.00034, and clustering coefficient in the range 0.709–0.863. No region-specific patterns are observed in the four network measures, although, depending upon the measure, networks of wells in some states have higher/lower values and networks in some other states have a good mix of high and low values. Further, Spearman rank correlation analysis shows that the four network measures do not have significant relationships with key statistical characteristics (mean, standard deviation, and coefficient of variation) of the groundwater levels. This indicates that the differences in the network measures cannot be explained only by the basic statistical properties of the groundwater level records.

## 1. Introduction

More than a quarter of the world’s available freshwater resources are constituted by groundwater. In recent decades, increasing population, improved living standards, inadequate freshwater supply, and advances in technology have contributed, in one way or another, to significant exploitation and depletion of groundwater resources. Groundwater is the only reliable source of safe drinking water in many countries; hence, its exploitation is a serious concern [1]. Issues such as climate change and environmental degradation are expected to increase the intensity of this problem in the future. Sustainable planning and management of groundwater resources, including the conjunctive use of surface water, is important for ensuring food, water, and energy security in the future. To achieve this, an in-depth understanding of groundwater flow and solute transport dynamics is required.

Groundwater systems are nonlinear dynamic systems that evolve in a complex manner and are often composed of a large number of interconnected components. In natural subsurface formations, the flow and transport of solutes are influenced by several factors, including the heterogeneity of the subsurface porous medium and the complex interactions of groundwater with surface water and atmospheric water, which occur at different spatial and temporal scales. The pioneering works during the 19th century and early 20th century [2,3,4,5] laid a strong foundation for studies on groundwater flow and transport. Following up on such studies, numerous attempts have been made during the past several decades to advance our understanding of groundwater flow and transport phenomena. Such attempts have led to the proposal of a wide variety of approaches and to the development and application of numerous mathematical models. The approaches include deterministic [6,7,8], stochastic [9,10], geostatistical [11,12], fractal-based [13,14,15], and data-driven [16,17] or machine-learning approaches [18,19].

Despite the progress made through such theoretical developments and applications to groundwater systems around the world, there remain numerous challenges; see [20,21] for some recent reviews of advances and challenges in groundwater modelling. Many of the key challenges in groundwater modelling and prediction are particularly associated with our lack of ability to properly represent the complexity and connectivity in groundwater systems.

In the context of complexity and connectivity, there have been some significant theoretical developments, in recent decades, in the field of complex systems science. Among these, complex network theory [22,23] is particularly important—a network is a set of points (or nodes) connected by a set of lines (or links). This theory is particularly useful for studying large, complex, nonlinear, and dynamically evolving systems. As a result, applications of the concepts of complex networks have been gaining momentum in hydrologic studies in recent years. Thus far, the theory has been used for studying a wide range of problems in hydrology, including spatial/temporal/spatio-temporal connections in rainfall and streamflow [24,25,26,27,28,29,30], catchment classification/regionalization [31], flood frequency analysis [32], optimal hydrologic monitoring networks [33], rainfall interpolation [34], the prediction of rainfall and streamflow [35,36], drought properties and propagation [37,38], and performance assessment of global climate models (GCMs) [39], among others. In the broader hydroclimatic context, complex network concepts have also been applied to other climate variables, such as pressure [40], integrated moisture flux [41], evaporation [42], surface air temperature [43,44], sea surface temperature [45,46,47], sea level pressure [45], and relative humidity [48], among others.

The outcomes of these studies indeed reveal that complex network theory offers unique perspectives on the complexity and connectivity in hydrologic systems. Despite this, applications of complex network theory have largely been limited to the domains of surface hydrology and hydroclimatology, and the theory has not gained much traction in studying groundwater systems. To our knowledge, only a very few studies have, thus far, applied the concepts of complex networks to study groundwater systems [49,50,51,52].

Sivakumar et al. [49] were the first to apply the complex network theory to the groundwater domain. They applied complex networks concepts to examine the spatial connections in daily groundwater levels observed in a monitoring network of 125 wells across California, USA. They used two complex network-based measures: (1) the clustering coefficient, which measures local density and quantifies the tendency of a network to form clusters; and (2) degree distribution, which measures the spread and fraction of nodes in a network with a specific number of links. They also used different correlation threshold values to identify the links and construct the groundwater-level network. Based on the high clustering coefficient values (even for high correlation thresholds), they suggested that the groundwater-level network was not a random graph and that it could be a small-world network. The degree distribution results revealed a combination of exponential distribution and power-law distribution, with significant resemblance to an exponentially truncated power-law distribution. Yu et al. [50] applied complex network theory to analyse the groundwater levels in the Nanxi Well in China, especially to study the impact of seismic actions on groundwater levels. Using the visibility graph (VG) and analysing both the raw groundwater level series and the trend groundwater level series, they reported that the nodes of high degrees were closely related to the seismic response of the well. They suggested that all of the VG networks were scale-free and hierarchical. They also reported that the seismic response of the groundwater level was reflected by degree correlation and that the community division of the raw VG network was basically the same as that of the trend VG network.

Rodríguez-Alarcón and Lozano [51] used complex network concepts to study both the spatial distribution and temporal evolution of groundwater wells in and around the Doñona Natural Space in Spain. They constructed an undirected weighted network from the wells. In addition to using several conventional complex network measures (e.g., density, strength, path length, transitivity, clustering coefficient, efficiency, centrality, assortativity), they proposed two specific abstraction-based centrality indexes: labelled ego impact (based on the total impact of a particular groundwater abstraction well on its neighbouring wells) and labelled alters impact (based on the total impact of surrounding wells on a specific location). The topology of the network revealed a large number of small, disconnected components and a few large components, which was also reflected in the power-law-type degree distribution. The network was also found to have a high average clustering coefficient, a small average path length, a low level of centralization, and high global and average local network efficiency. The network was assortative in terms of degree–degree and strength–strength correlations, and the eigenvector centrality of the nodes revealed the location of influential wells. Bharti et al. [52] applied the transfer entropy coupled with directed-weighted complex network for analysis of groundwater levels over the period 1990–2009 at 133 wells in the Sina River basin in Maharashtra, India. They used the canopy clustering method and identified five clusters in the 133 wells. They employed the transfer entropy to obtain the direction and weight of the links of the groundwater-level network. They studied seasonal, pre-monsoon and post-monsoon data. They determined the directional relationships between selected wells from different clusters and also identified the most influenced well using node strength and directed clustering coefficients. They also reported, based on clustering coefficients for all clusters, that all wells within a cluster formed clusters among themselves, though the strengths of the clusters were found to vary.

Encouraged by the outcomes of the above studies, the present study applies complex network theory to examine the temporal dynamics of groundwater levels across a large region. The study introduces, in the groundwater domain, a new approach to construct the temporal network of groundwater levels, by coupling chaos theory with complex network theory for network formation, following up on the approach originally proposed by Yasmin and Sivakumar [28] for streamflow. The present study is the first ever instance in which this coupled chaos theory–complex network framework is applied for studying groundwater-level dynamics, and in the groundwater domain more broadly. Furthermore, unlike previous studies on the application of complex network theory to groundwater, which mainly examined connections between/among different wells in a spatial network sense, this study constructs a temporal network for each well based on the corresponding groundwater level records. In the present study, the coupled framework is applied to daily groundwater-level data recorded during 2010–2014 at each of 71 wells across the United States. This large-scale application allows the complexity and temporal connectivity of groundwater-level records from different regions to be compared using a common framework.

When studying temporal dynamics, each well is considered as a network. The phase space reconstruction technique [53,54] is used to reconstruct the single-variable groundwater time series in a multi-dimensional phase space. The optimum dimension required for the phase-space reconstruction is determined using the false nearest neighbour (FNN) algorithm [55]. Each reconstructed vector in the phase space is considered as a node in the network, and the connections between the nodes (i.e., links) are identified based on the distance threshold between the reconstructed vectors. Four complex network-based measures—degree centrality, betweenness centrality, closeness centrality, and clustering coefficient—are used to analyse the properties of the groundwater-level network. Degree centrality identifies the number of connections a node has, highlighting its central or influential role within the network. Betweenness centrality measures the extent to which a node acts as a bridge in the network. Closeness centrality evaluates the efficiency of information transfer across the network, through determining how close a node is to the other nodes in the network. Clustering coefficient measures the extent to which nodes in the network form tightly connected groups. In addition, Spearman rank correlation analysis is performed to examine the relationships between the four network measures and key statistical characteristics (mean, standard deviation, and coefficient of variation) of the groundwater-level records. This comparison helps determine whether the network measures provide information beyond the basic statistical properties of the groundwater-level data.

The rest of this manuscript is organized as follows. Section 2 presents the methodology, including brief descriptions of network, network formation for groundwater-level data in a temporal perspective, and the four network measures used in this study. Section 3 describes the study area and groundwater-level data used for analysis. The results and discussion are presented in Section 4. Conclusions, limitations, and future directions are presented in Section 5.

## 2. Methods

### 2.1. Network and Construction

A network consists of a collection of nodes, also known as vertices, and the connections between them, known as links or edges. Mathematically, a network can be expressed as G = {P, E}, where P represents the set of nodes and E is a set of edges connecting them. In the context of the present study, let us consider a groundwater-level record observed at a daily temporal resolution for a particular well over a specified period, denoted by *X_i_*, where *i* = 1, 2, 3, …, *N*. To construct a temporal network from this groundwater-level series, each observation time (i.e., daily), can be treated as an individual node. Based on this, it can be assumed that a link between any two nodes is established when the difference between their corresponding groundwater-level values is smaller than a predefined threshold. If the difference exceeds the threshold, the two nodes are considered unconnected.

In this study, the temporal network for the groundwater-level data is constructed by coupling chaos theory and network theory, following the study by Yasmin and Sivakumar [28] for streamflow data. First, the one-dimensional groundwater level time series, *X_i_*, is constructed in a multi-dimensional phase space using the concept of phase-space reconstruction [53,54] as follows:

***Y****_j_* = (*X_j_*, *X_j_*_+*τ*_, *X_j_*_+2*τ*_,…, *X_j_*_+(*m*−1)*τ*_) (1)

where *j* = 1, 2, …, *N* − (*m* − 1)*τ*/∆*t*, *m* is the embedding dimension of the vector, and *τ* is a delay time. Several methods are available for selecting a suitable embedding dimension (*m*) that can adequately represent the underlying system dynamics. This dimension is commonly referred to as the optimum embedding dimension (*m_opt_*). In this study, the false nearest neighbour (FNN) algorithm, originally proposed by Kennel et al. [55], is employed. The basic idea of the FNN method is to examine whether points that appear to be close to each other in an *m*-dimensional reconstructed phase space remain close when the dimension is increased to *m +* 1. For each phase-space vector ***Y****_j_*, its nearest neighbor $Y_{j}^{\mathrm{NN}}$ is identified in dimension *m.* If this neighbour remains close to ***Y****_j_* after increasing the embedding dimension, then it is considered a true neighbor. However, if the neighbor moves far away when the dimension is increased, then the point is identified as a false neighbor. As the embedding dimension is gradually increased, the number of false nearest neighbors would decrease. The optimum dimension is selected as the dimension at which the percentage of false nearest neighbors becomes zero. In the FNN algorithm, two threshold criteria are used to decide whether a neighbor is false or true. These criteria are loneliness tolerance and distance tolerance; see, for example, Sangoyomi et al. [56] for details.

After the phase-space reconstruction, each reconstructed vector is considered as a node of the network. The links between the nodes are identified based on the Euclidean distance between the reconstructed vectors. A pair of nodes is connected if the Euclidean distance between them is smaller than a selected threshold, whereas no link is assigned if the distance exceeds this threshold. The threshold is identified by determining the network density, which is the ratio of the actual number of connections in the network to the maximum possible number of connections in the network; for details on the selection of different distance thresholds and the threshold at which the slope of the network density is maximum, see Deepthi and Sivakumar [39].

### 2.2. Network Measures

There exist many different measures and methods to identify the properties of a network. In the present study, four different network measures are used: degree centrality, betweenness centrality, closeness centrality, and clustering coefficient. These measures are briefly described next.

#### 2.2.1. Degree Centrality

Degree centrality (DC) identifies the influence of a node in a network based on degree, which is the number of connections a particular node has. It is useful to identify whether a given node is more central or influential than another node. For a network with *N* nodes, the degree centrality of a given node *j* is the ratio of the number of “actual neighbours” of node *j* to the number of possible neighbours of node *j*, and is given as

(2) $${\mathrm{DC}}_{j}=\frac{\sum_{k=1}^{N}A_{j,k}}{N-1},$$

where A_j,k_ denotes the symmetric adjacency matrix, with A_j,k_ = 1 when a link exists between the *j*th and *k*th nodes and A_j,k_ = 0 when there no link exists between them.

#### 2.2.2. Betweenness Centrality

Betweenness centrality (BC) measures how many times a node lies in the shortest path between the other nodes in a network [57]; the shortest path between any given pair of nodes is the path with the minimum number of links. Therefore, the BC of a node measures its ability, by acting as a bridge with the other nodes, to transfer the information within a network. The BC of node *j* in a network is given by

(3) $${\mathrm{BC}}_{j}=\sum_{k\neq l\neq j\in \left\{N\right\}}^{N}\frac{\sigma_{j}\left(k,l\right)}{\sigma \left(k,l\right)},$$

where *σ*(*k*,*l*) is the total number of shortest paths between the node pairs *k* and *l*; and *σ_j_*(*k*,*l*) indicates the total number of shortest paths between the node pairs *k* and *l* passing through the node *j*.

#### 2.2.3. Closeness Centrality

Closeness centrality (CC) measures how close a node is to the remaining nodes in a network. It estimates how long it will take to spread the information within the network. The CC of a node is the inverse of the sum of all shortest paths to the other nodes in the network. The CC of node *j* is given by

(4) $${\mathrm{CC}}_{j}=\frac{1}{\sum_{k=1}^{N}d_{\mathrm{jk}}}\;,$$

where *d_jk_* is the shortest path between the node pairs *j* and *k*.

#### 2.2.4. Clustering Coefficient

Clustering coefficient (C_c_) quantifies the tendency of nodes in a network to cluster together [22]. Let us consider a node *j* in a network having connections with *m* other nodes; i.e., node *j* has *m_j_* links. If the neighbours of node *j* were a part of a cluster, then the possible number of links among *m_j_* nodes would be $\frac{m_{j}(m_{j}-1)}{2}$. The clustering coefficient of node *j* is the ratio of the actual links (*E_j_*) that exists between these *m_j_* nodes to the possible number of links, and is given by

(5) $$C_{c_{j}}=\frac{2E_{j}}{m_{j}(m_{j}-1)},$$

### 2.3. Statistical Analysis

Statistical analysis is performed to determine whether the differences in the network measures are related to the basic statistical properties of the groundwater-level records. For this purpose, Spearman rank correlation analysis is used to examine the relationships between some key groundwater-level characteristics and the network measures across the 71 wells. The mean, standard deviation, and coefficient of variation of the groundwater levels are correlated with the mean degree centrality, betweenness centrality, closeness centrality, and clustering coefficient. Spearman’s correlation coefficient (*ρ*) ranges from −1 to +1. A positive value of *ρ* indicates that the groundwater-level statistic and the network measure tend to increase together. A negative value indicates that the network measure tends to decrease as the groundwater-level statistic increases, or vice versa. A value close to zero indicates no monotonic relationship between them.

The *p*-value is used to assess the statistical significance of each correlation (i.e., between groundwater statistic and network measure). Four network measures are correlated with three statistical properties of the groundwater levels, resulting in a total of 12 correlations. The raw *p*-value represents the significance of each correlation before correction. As several correlations are tested, some may appear statistically significant simply by chance. Therefore, the raw *p*-values are adjusted using the Benjamini–Hochberg false-discovery-rate (FDR) procedure [58]. The adjusted *p*-value represents the significance level after accounting for these multiple comparisons and is used to determine the final statistical significance. A relationship is considered statistically significant when its FDR-adjusted *p*-value is less than 0.05.

All of the computations in this study and the generation of the figures were performed using MATLAB R2024b and Python 3.9.6.

## 3. Study Area and Data

**Figure 1:**
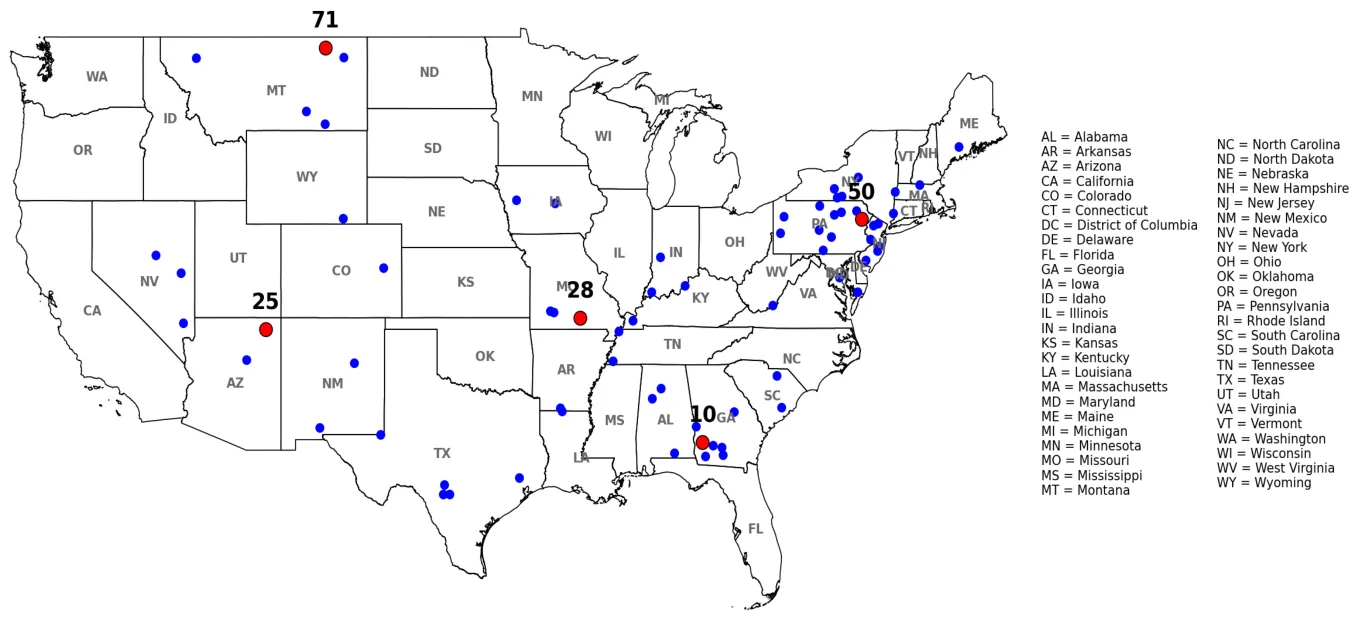
Locations of the 71 groundwater wells in the United States considered in this study. The five wells that are chosen for the purpose of illustration of the analysis and results are marked in red. These wells are (1) Well 28 (No. 365949091421401) at Howell, MO; (2) Well 10 (No. 313953084361202) at Randolph, GA; (3) Well 50 (No. 411223075234901) at Monroe, PA; (4) Well 71 (No. 483148106281601) at Valley, MT; and (5) Well 25 (No. 362936109564101) at Apache, AZ. See Table 1 for further details.

In the present study, temporal dynamics of groundwater levels in the United States are examined using complex network theory. Specifically, daily groundwater-level data observed at 71 wells across the contiguous United States are studied. Figure 1 shows the locations of the 71 wells considered in this study. The five wells that are marked in red are chosen for the purpose of illustration of the analysis and results; more details on this are provided later.

The 71 wells considered in this study are geographically distributed across the contiguous United States and represent a wide range of climatic zones, hydrogeologic settings, and groundwater use regimes. The study area also spans key physiographic regions of the country, including the Great Plains, Central Valley of California, the Mississippi Embayment, the Columbia Plateau, and the Atlantic Coastal Plain. Each region exhibits unique groundwater characteristics driven by differences in geology, precipitation, land use, and human intervention.

Several of the 71 wells considered in this study fall within the High Plains Aquifer, one of the most heavily used aquifer systems in the United States, extending from Texas to South Dakota. This aquifer consists mainly of unconsolidated sand and gravel deposits and supports intensive irrigation across semi-arid regions. Groundwater levels in this aquifer have declined significantly in recent decades due to persistent over-extraction [59]. In the Central Valley Aquifer of California, the wells selected for this study are located in one of the most productive agricultural areas in the world. The aquifer consists of thick sequences of alluvial deposits and experiences high seasonal and interannual variability in groundwater levels. Groundwater overdraft has been a long-standing concern in this region, particularly during periods of drought [60].

The Floridan Aquifer System, represented by wells in the southeastern United States, is a major carbonate aquifer that supplies water to Florida and parts of Georgia, Alabama, and South Carolina. Its high transmissivity and karstic features result in complex flow patterns and strong connections between surface and groundwater systems [61]. Coastal areas within this system face increasing vulnerability to saltwater intrusion due to sea-level rise and excessive pumping. Wells selected along the Northern Atlantic Coastal Plain Aquifer System span areas of the Mid-Atlantic and southeastern US coastline. These aquifers are composed of layered unconsolidated sediments and are susceptible to both overuse and saltwater intrusion, particularly in rapidly urbanizing coastal zones [62]. In the Pacific Northwest, wells located within the Columbia Plateau Aquifer System are influenced by thick basalt flows interbedded with sedimentary units. Recharge in this region is driven largely by snowmelt and localized precipitation, and groundwater is a critical resource for irrigation and public supply in eastern Washington and Oregon [63]. The Mississippi Embayment region, including parts of Arkansas, Mississippi, and Tennessee, contains thick alluvial aquifers used primarily for irrigation. Groundwater in this region is closely tied to the Mississippi River system, with surface–subsurface exchanges playing an important role in local water balance.

Across the study area, the selected wells are located near major river systems, such as those of the Mississippi, Colorado, Columbia, Missouri, and Sacramento–San Joaquin rivers. These rivers are essential for recharge processes, especially in semi-arid and arid regions, and often intersect major alluvial aquifers. The integration of surface water features with well locations enables a more complete understanding of regional hydrologic connectivity and groundwater dynamics [64].

The daily groundwater-level data for the period 2010–2014 are downloaded from the US Geological Survey (USGS) National Water Information System (NWIS) (https://dashboard.waterdata.usgs.gov/app/nwd/en/ (accessed on 10 March 2026)). Table 1 presents some basic details of the 71 groundwater wells considered in this study, including the well number, location (latitude, longitude, county, and state), as well as the statistics of the groundwater-level data, including mean, standard deviation, and coefficient of variation (CV). The groundwater-level data from the 71 wells exhibit enormous variations, such as (a) a mean level that ranges from 2.62 ft/day (at Hunterdon, New Jersey) to 431.90 ft/day (at White Pine, Nevada); (b) a standard deviation that ranges from 0.13 ft/day (at Apache, Arizona) to 35.25 ft/day (at Medina, TX); and (c) a coefficient of variation that ranges from 0.01 (at several wells) to 0.68 (at Hunterdon, New Jersey).

**Figure 2:**
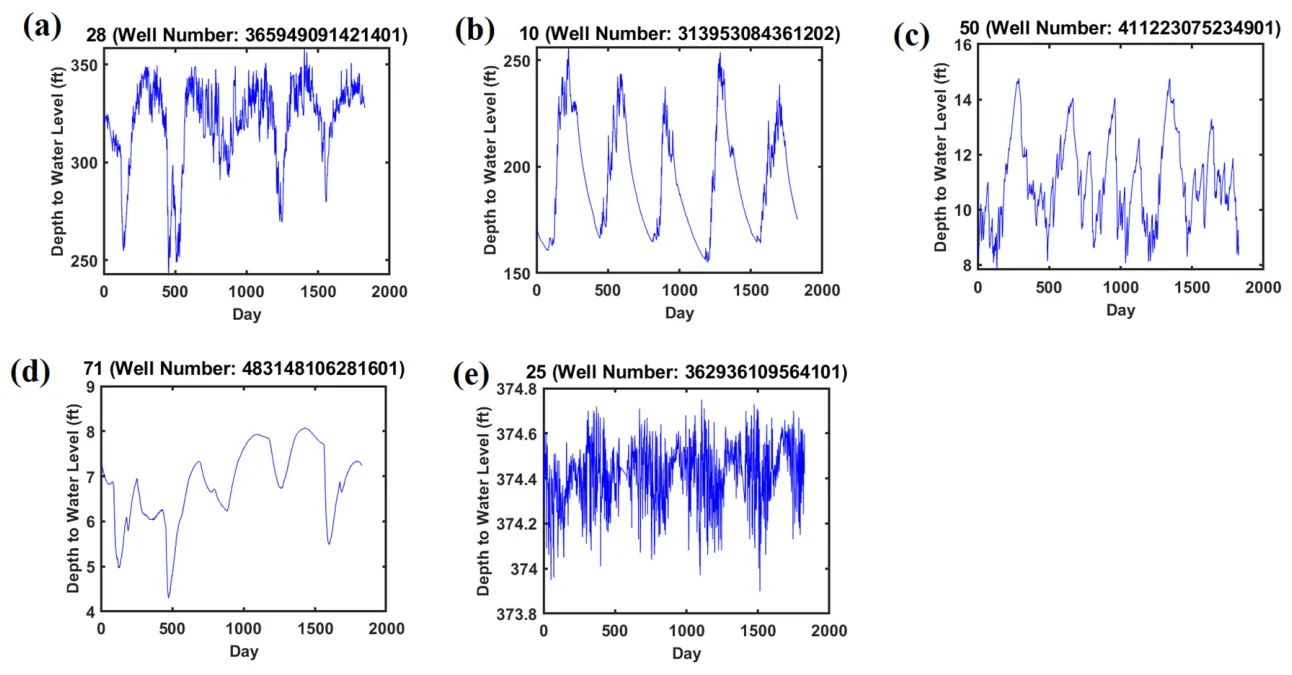
Time series of groundwater-level data from five selected wells: (**a**) Well 28 (Howell, MO); (**b**) Well 10 (Randolph, GA); (**c**) Well 50 (Monroe, PA); (**d**) Well 71 (Valley, MT); and (**e**) Well 25 (Apache, AZ).

Figure 2a–e presents the time series plots of the daily groundwater levels observed over the period 2010–2014 at five of the 71 wells considered in this study. These five wells are considered here for the purpose of illustration of the analysis and results. These wells, according to serial numbers in Table 1, are (a) Well 28 (No. 365949091421401) at Howell, MO; (b) Well 10 (No. 313953084361202) at Randolph, GA; (c) Well 50 (No. 411223075234901) at Monroe, PA; (d) Well 71 (No. 483148106281601) at Valley, MT; and (e) Well 25 (No. 362936109564101) at Apache, AZ. As seen, the daily groundwater levels at each of these five wells exhibit significant temporal variability and distinct patterns as well, but with certain seasonal variability common to all, as may be expected.

## 4. Results

### 4.1. Network Construction

**Figure 3:**
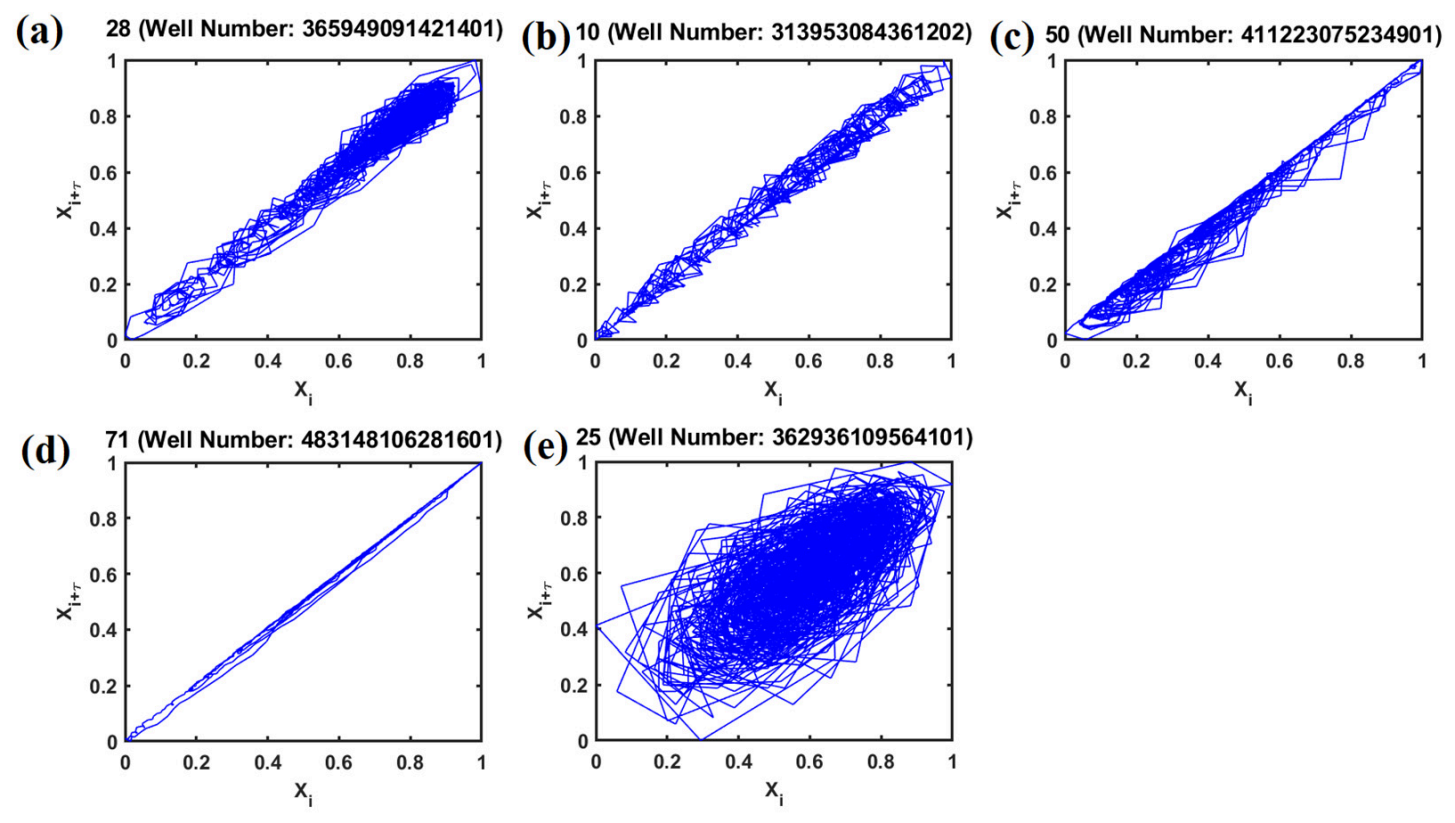
Phase-space plots of groundwater-level data from five selected wells: (**a**) Well 28 (Howell, MO); (**b**) Well 10 (Randolph, GA); (**c**) Well 50 (Monroe, PA); (**d**) Well 71 (Valley, MT); and (**e**) Well 25 (Apache, AZ).

Each of the 71 wells is considered as a network. The groundwater level time series from each of these wells is normalized to the range of 0 to 1. The phase space is reconstructed with a delay time value of 1, which is also used in the subsequent FNN algorithm to determine the optimal embedding dimension. Figure 3a–e shows the two-dimensional phase-space diagrams for the groundwater level time series over the period 2010–2014 from the five selected wells mentioned above (Wells 28, 10, 50, 71, and 25). As seen, the trajectories of the groundwater-level data exhibit somewhat different patterns. The trajectories for the first four time series occupy only a narrow region (Figure 3a–d), perhaps suggesting less complexity; nevertheless, minor variations among them are also seen. On the other hand, the trajectory for the fifth time series (Figure 3e (Well 25, from Apache, AZ)) occupies a considerably large region in the phase space and more scattered, perhaps suggesting greater level of complexity. The phase-space plots for the other 66 groundwater level time series also similarly indicate different patterns and levels of complexity, as may be the case. These observations suggest, at least in a qualitative sense, that the groundwater level time series from the 71 wells exhibit a low, medium, or high level of complexity, depending upon the time series.

The FNN analysis is performed with embedding dimensions from 1 to 20. The embedding dimension that yields zero, or the lowest, percentage of false nearest neighbours (%FNN) is identified as the optimum embedding dimension (*m_opt_* or FNN dimension). If more than one embedding dimension gives the lowest %FNN, then the lesser/least between/among those dimensions is considered as the optimum dimension. The phase-space reconstructed vectors corresponding to the optimal embedding dimension are then used for the construction of the groundwater-level network.

**Figure 4:**
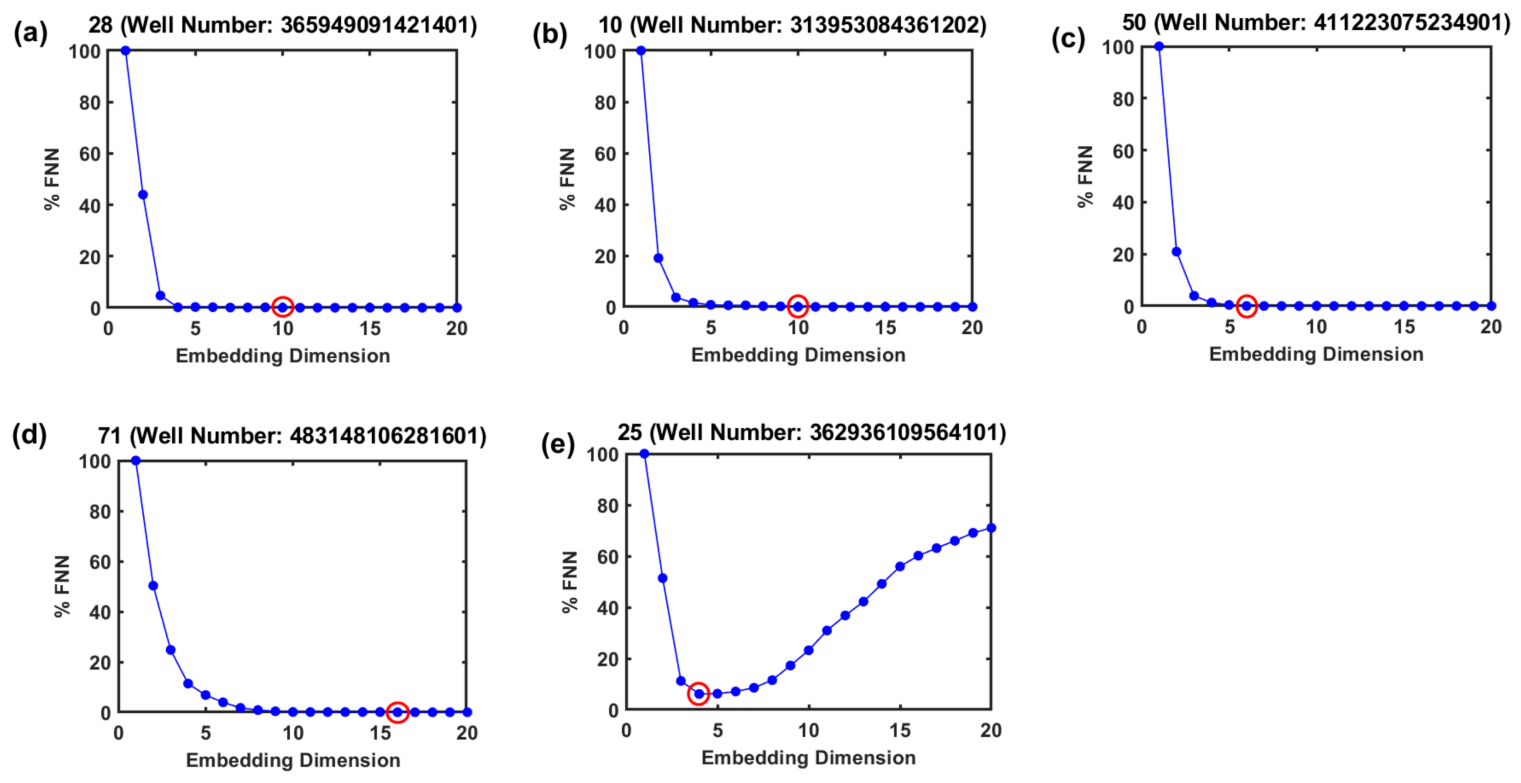
False nearest neighbour (FNN) plots of groundwater-level data from five selected wells: (**a**) Well 28 (Howell, MO); (**b**) Well 10 (Randolph, GA); (**c**) Well 50 (Monroe, PA); (**d**) Well 71 (Valley, MT); and (**e**) Well 25 (Apache, AZ). The embedding dimensions marked with red circles indicate the optimum embedding dimensions (*m_opt_*).

Figure 4a–e shows the FNN dimension plots for the groundwater level time series from the five wells (28, 10, 50, 71, and 25). As seen, the %FNN value initially decreases with an increase in the embedding dimension up to a certain point and then either saturates (as in the case of the first four series; Figure 4a–d) or again increases (as in the fifth series, Figure 4e) when the embedding dimension is increased further. It is also important to note that, in some cases, after an initial steep fall in the %FNN at very low embedding dimensions, the %FNN may not completely reach saturation at the immediate higher embedding dimensions, but only fall very gradually before reaching saturation at a still higher embedding dimension (or may even continue to decrease), as is the situation for Wells 28, 10, and 71 (Figure 4a,b,d). Therefore, it may sometimes be difficult to identify the optimal embedding dimension. In the present study, the optimum embedding dimension is identified as the embedding dimension corresponding to the minimum percentage of false nearest neighbours (FNNs). According to this, the optimal embedding dimensions for Wells 28, 10, 50, 71, and 25 are identified as 10, 10, 6, 16, and 4, respectively.

**Figure 5:**
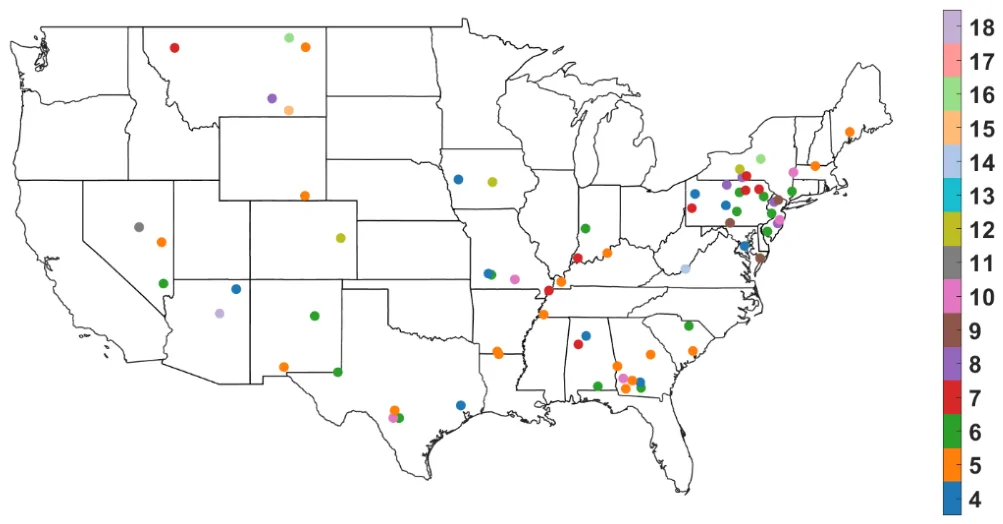
Optimal embedding dimensions (*m_opt_*) obtained from the false nearest neighbour (FNN) algorithm for groundwater level time series from all the 71 wells.

Figure 5 shows the optimal embedding dimensions (i.e., *m_opt_* or FNN dimensions) obtained for the 71 groundwater level time series using the FNN method; see also Table 2. Overall, the optimal embedding dimensions range from as low as 4 (for nine wells) to as high as 18 (for one well), suggesting a wide range of complexity in the groundwater level time series. For most of the time series (62 out of 71; about 88%), the optimal embedding dimensions are found to be in the range of 4–10, suggesting the low-to-medium-level complexity of the groundwater level dynamics. Optimal embedding dimensions greater than 10 are observed only for nine of the 71 groundwater level series (with a maximum dimension of 18), which may be considered to indicate a higher level of complexity. The FNN dimension results also indicate significant variability in the groundwater levels across wells in some states; for instance, the groundwater levels in the five wells in Montana (MT), i.e., Wells 67, 68, 69, 70, and 71, have optimal dimensions of 15, 8, 7, 5, and 16, respectively, indicating a wide range of complexity. Similar observations are also made for the groundwater levels across wells in Arizona (AZ), Pennsylvania (PA), New York (NY), Iowa (IA), and Georgia (GA), among others. On the other hand, groundwater levels across wells in some other states, such as New Mexico (NM), Alabama (AL), South Carolina (SC), and Indiana (IN), have somewhat similar levels of complexity, especially lower level of complexity, with *m_opt_* ≤ 6.

Each of the 71 groundwater level time series is now reconstructed with the corresponding optimal embedding dimension. For each series, the reconstructed vectors are considered as the nodes of the temporal groundwater network and the connections among them are considered the links. The identification of the existence of links between any pair of nodes is made based on the threshold distances, which are assumed in accordance with the Euclidean distances between the reconstructed vectors. The optimum or critical distance threshold (*DT_c_*) is identified by calculating the network density (i.e., the ratio of the actual number of links to the maximum possible number of links in the network) for different thresholds and determining the threshold at which the slope of network density is maximum. The critical distance thresholds obtained for the 71 groundwater level time series are presented in Table 2. For each of the 71 series, the temporal network is formed based on the critical distance threshold, and the four network measures are calculated.

### 4.2. Network Measures Results

#### 4.2.1. Degree Centrality Results

**Figure 6:**
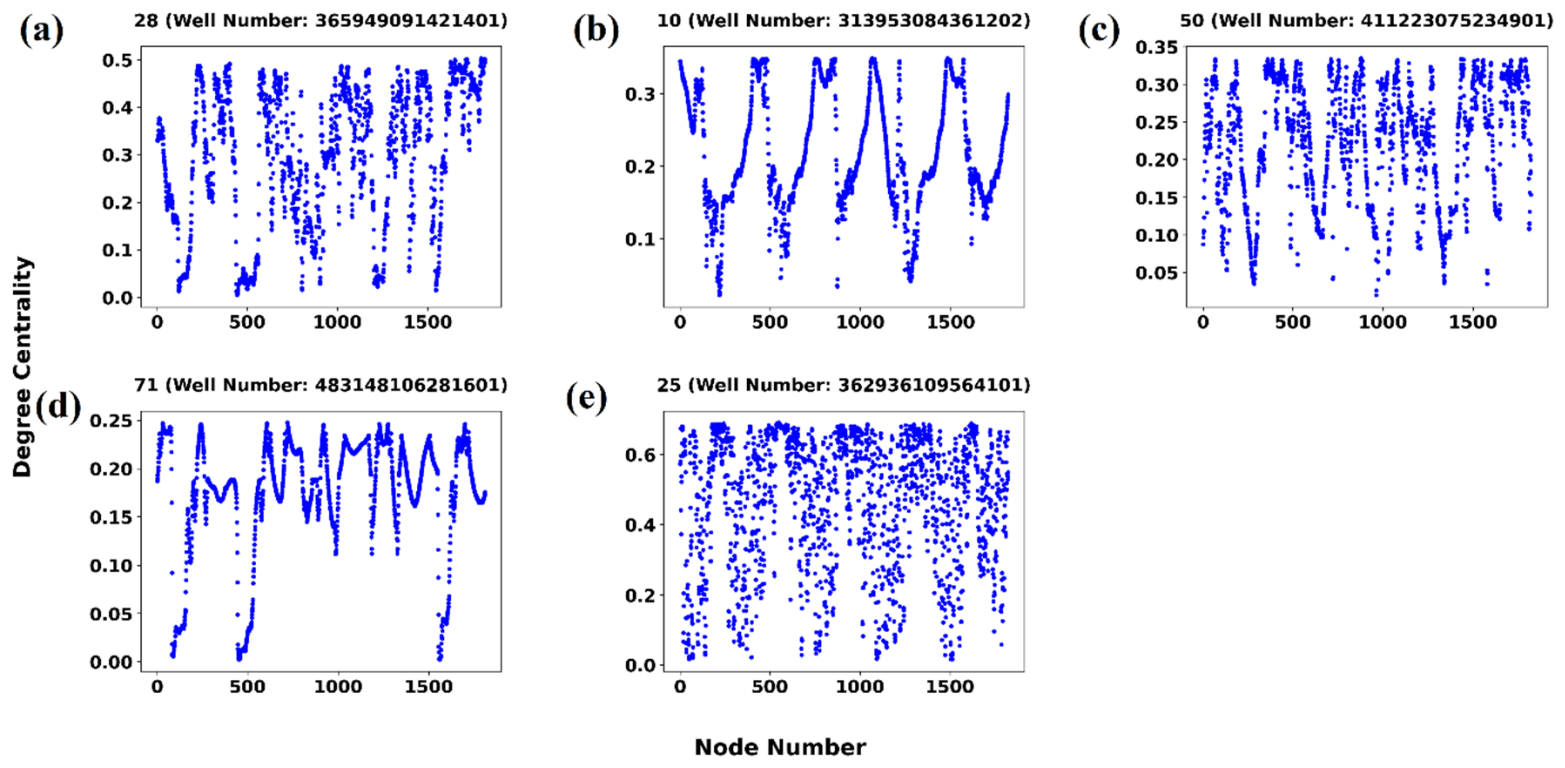
Degree centrality values for groundwater-level networks from five selected wells: (**a**) Well 28 (Howell, MO); (**b**) Well 10 (Randolph, GA); (**c**) Well 50 (Monroe, PA); (**d**) Well 71 (Valley, MT); and (**e**) Well 25 (Apache, AZ).

Figure 6a–e shows the degree centrality (DC) values obtained for the different nodes in each of the five selected groundwater-level networks, i.e., Wells 28, 10, 50, 71, and 25. For each of the five networks, the DC values indicate that different nodes may have different connectivity levels; in some cases, the connectivity levels of the nodes span a very wide range. The ranges of the DC values of nodes for the five networks are Well 28: 0.004–0.503; Well 10: 0.022–0.349; Well 50: 0.020–0.335; Well 71: 0.002–0.248; and Well 25: 0.015–0.690. The mean DC values for the networks from Wells 28, 10, 50, 71, and 25 are 0.292, 0.221, 0.223, 0.174, and 0.465, respectively. These values suggest that the overall influence of nodes of the groundwater network from Well 25 (Apache, AZ) (DC = 0.465) is much higher than that of the other four networks. The overall influence of nodes of the network from Well 71 (Valley, MT) (DC = 0.174) is the lowest. Further, the influence of nodes of the network from Well 28 (Howell, MO) is slightly higher when compared with the other three (Wells 10, 50, and 71).

The DC values also suggest that some nodes have a significantly higher number of direct connections with the other nodes, perhaps indicating their considerable influence in the network. These nodes also seem to suggest the role of seasonality on the network connectivity, as certain seasonal patterns are observed in the DC values. In general, for each of the above five networks, there are only a very few nodes with very low connectivity. For Well 28 (Howell, MO) (Figure 6a) and Well 25 (Apache, AZ) (Figure 6e), which generally have high connectivity levels, there is a relatively more even distribution of connectivity of the nodes. Well 50 (Monroe, PA) (Figure 6c) also has a relatively more even distribution of connectivity of the nodes, though the range of DC values in which the distribution is even is not significant. For Well 71 (Valley, MT) (Figure 6d), most of the nodes have relatively higher connectivity levels, with only very few nodes having connectivity levels close to zero. A somewhat similar situation is also observed for Well 10 (Randolph, GA) (Figure 6b), but with a slightly larger number of nodes having intermediate connectivity levels.

**Figure 7:**
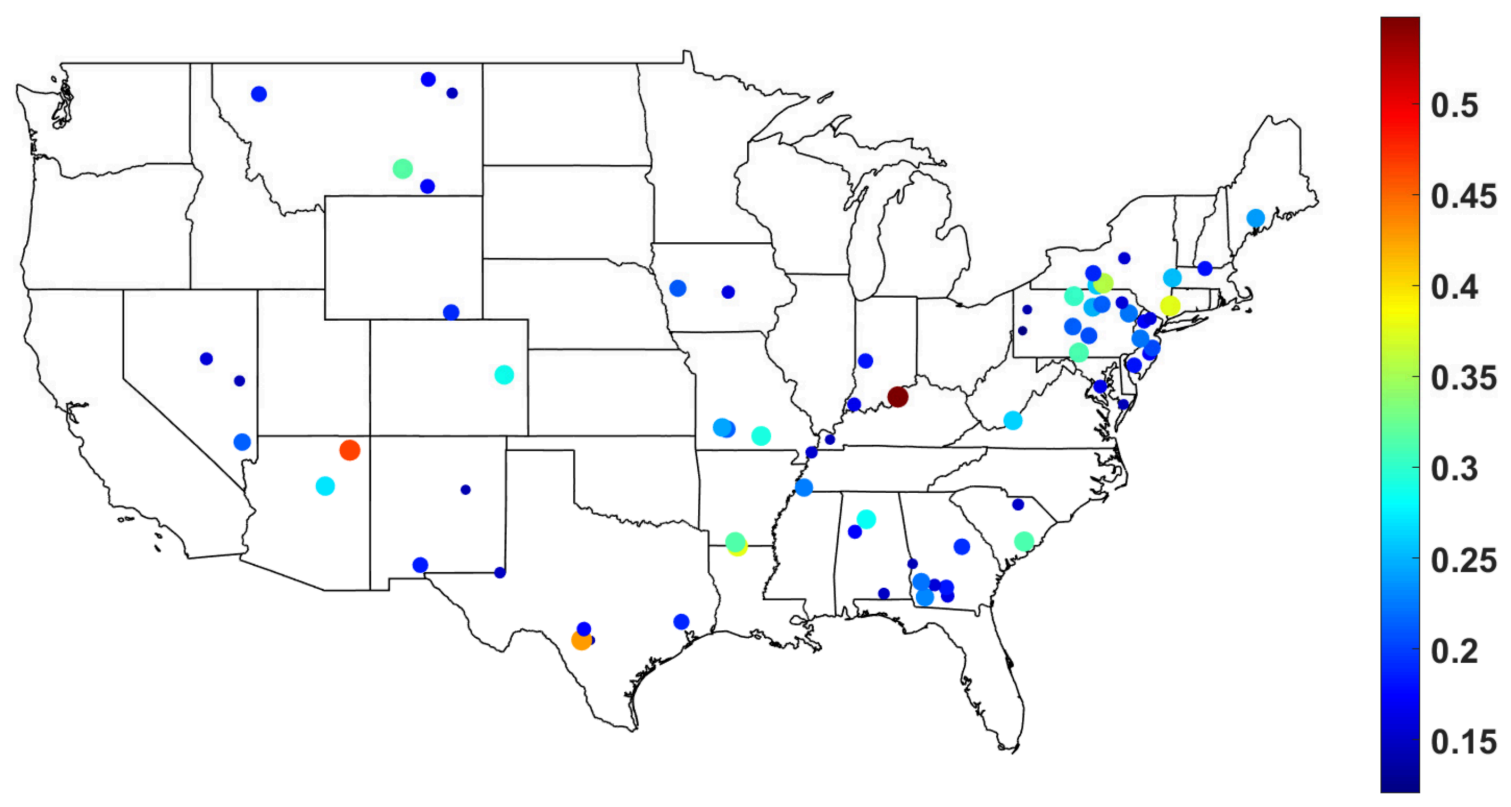
Degree centrality results for groundwater-level networks from all of the 71 wells for the optimal distance thresholds.

Figure 7 presents the degree centrality values obtained for the 71 groundwater-level networks. Considering all of the 71 groundwater-level networks, the degree centrality values are in the range 0.12–0.55 (Table 2). The highest DC value of 0.55 is obtained for Well 34 (Clark, IN), followed by a DC value of 0.46 obtained for Well 25 (Apache, AZ). The lowest DC value of 0.12 is obtained for Well 46 (Allegheny, PA), closely followed by 0.14 obtained for six wells, including Well 1 (Medina, TX). Ten wells have DC values greater than or equal to 0.30, while 14 wells have DC values less than or equal to 0.15. There are no specific patterns in the DC values of groundwater-level networks from a regional perspective, although networks of wells in some southwestern states (e.g., AZ) have higher DC values and those in some states in the west/southwest (e.g., NV and NM) have lower DC values. Networks in some states (e.g., TX, PA, and IN) have a good mix of high and low DC values.

#### 4.2.2. Betweenness Centrality Results

**Figure 8:**
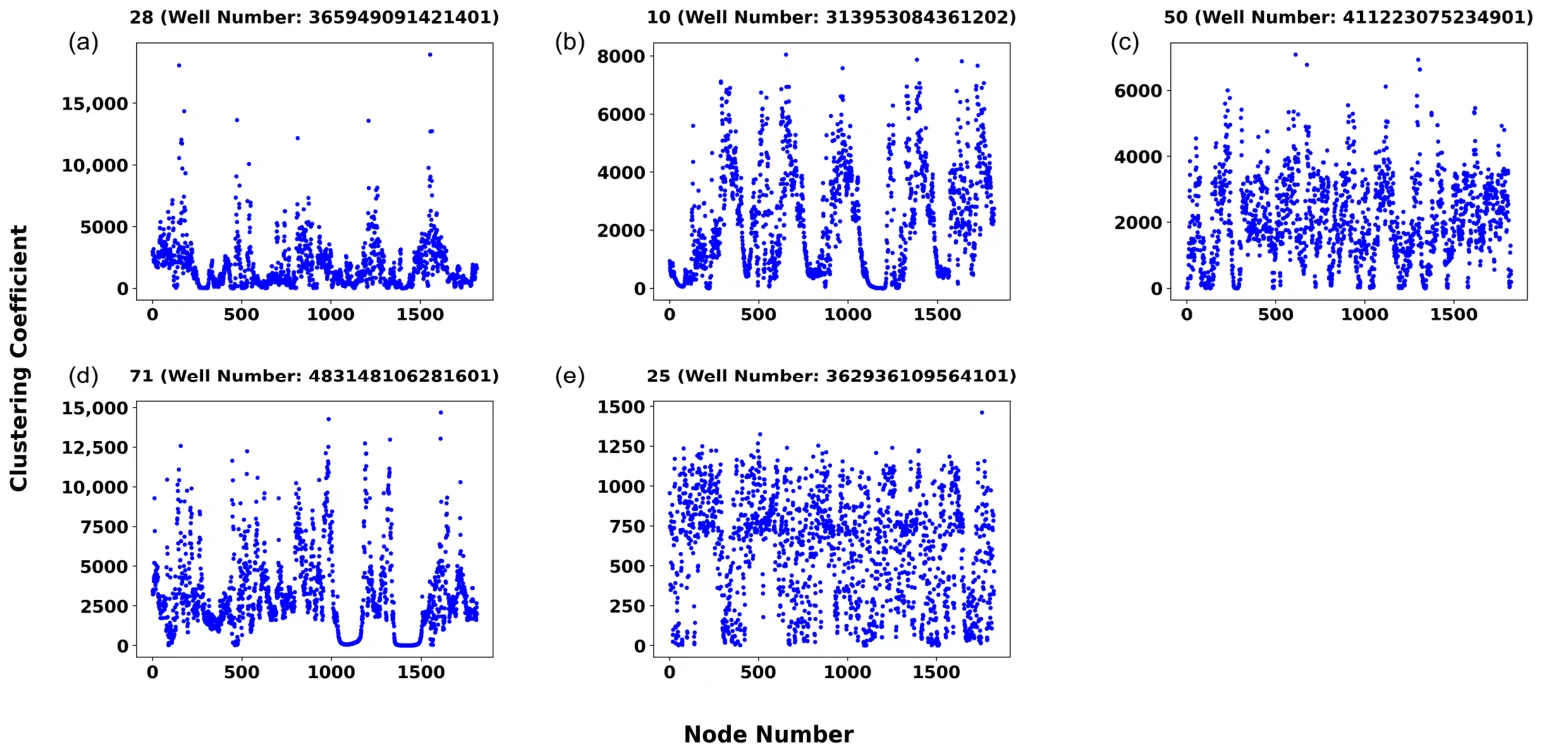
Betweenness centrality values for groundwater-level networks from five selected wells: (**a**) Well 28 (Howell, MO); (**b**) Well 10 (Randolph, GA); (**c**) Well 50 (Monroe, PA); (**d**) Well 71 (Valley, MT); and (**e**) Well 25 (Apache, AZ).

The betweenness centrality (BC) values obtained for the different nodes in each of the five selected groundwater-level networks, i.e., Wells 28, 10, 50, 71, and 25, are shown in Figure 8a to e, respectively. Similar to the observations made with the DC values, the BC values indicate different connectivity levels for different nodes. The ranges of the BC values of nodes for these networks span a very wide range, with 0.819–18936 for Well 28, 0–8044 for Well 10, 0–7080 for Well 50, 0–14677 for Well 71, and 0.336–1460 for Well 25. The mean BC values for these five networks are 1700, 2286, 2022, 3072, and 604, respectively. These BC values suggest that the groundwater network from Well 71 (Valley, MT) (BC = 3072) has a much greater overall ability, by acting as a bridge with the other nodes, to transfer information among the nodes. This is particularly the case when compared with that of the network from Well 25 (Apache, AZ), which has a BC value as low as 604. The ability of the other three networks to transfer information among the nodes is in between these two networks. Among these three networks, the networks from Well 10 (Randolph, GA) (BC = 2286) and Well 50 (Monroe, PA) (BC = 2022) have somewhat similar ability and are noticeably different from that of Well 28 (Howell, MO) (BC = 1700). For the networks from Well 28 (Howell, MO) (Figure 8a) and Well 50 (Monroe, PA) (Figure 8c), only a very few nodes have very high BC values. For the network from Well 25 (Apache, AZ) (Figure 8e), the nodes have significantly more even distribution of BC values. For the networks from Well 10 (Randolph, GA) (Figure 8b) and Well 71 (Valley, MT) (Figure 8d), the situation is somewhat in between the above two cases.

**Figure 9:**
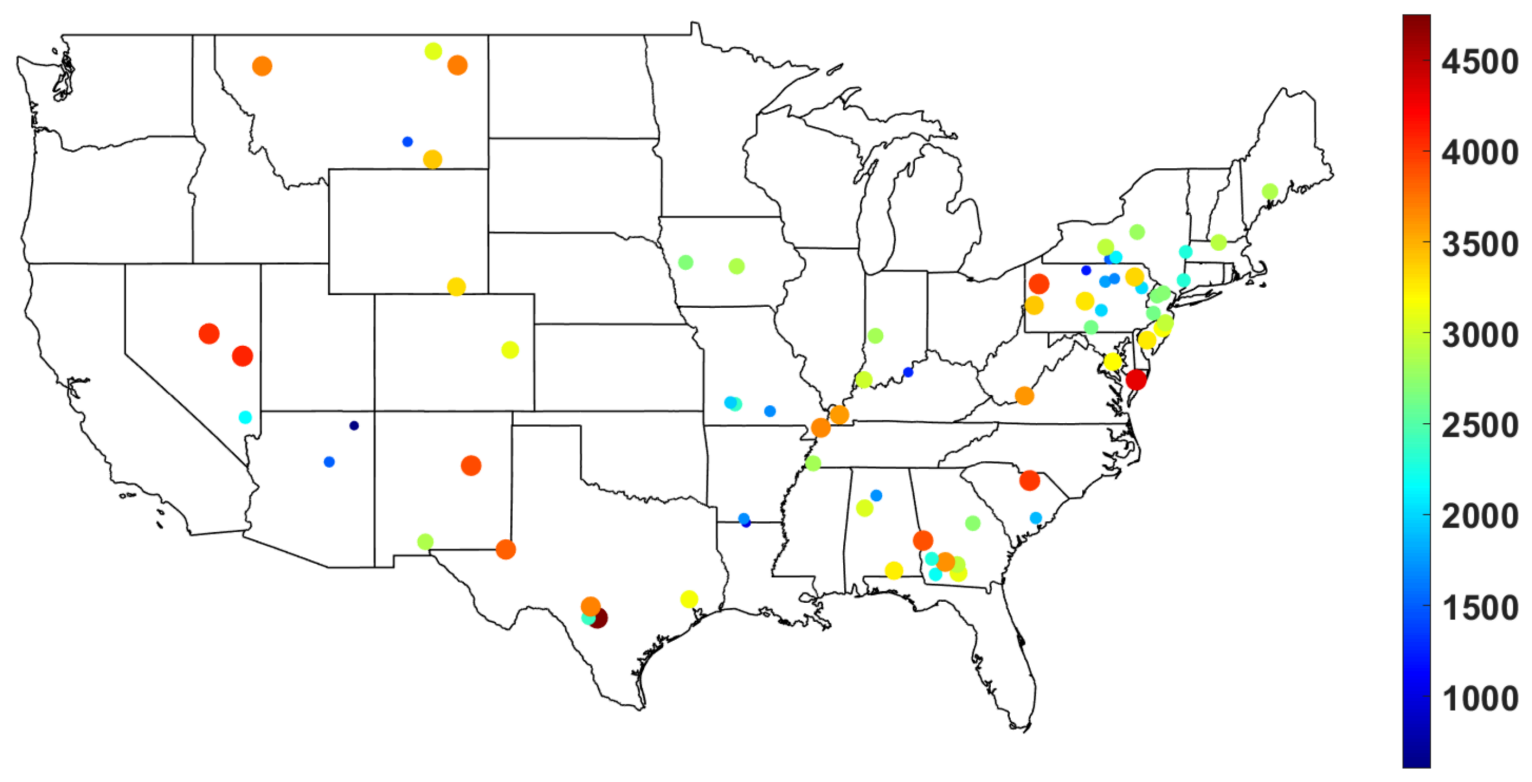
Betweenness centrality results for groundwater-level networks from all the 71 wells for the optimal distance thresholds.

Figure 9 shows the BC values obtained for the 71 groundwater-level networks. The betweenness centrality values are in the range 604–4750 (Table 2). The highest BC value of 4750 is obtained for the network from Well 1 (Medina, TX), followed by BC value of 4322 obtained for the network from Well 32 (Somerset, MD). The lowest BC value of 604 is obtained for the network from Well 25 (Apache, AZ), followed by a significantly higher value of 1206 for the network from Well 57 (Potter, PA) and 1264 for the network from Well 34 (Clark, IN). Thirty wells have BC values greater than 3000, while 14 wells have BC values less than 2000. There are no regional patterns in the BC values of groundwater-level networks. However, networks of wells in some northwest, west, and southwest states (e.g., MT, NV, and TX) and southeastern states (e.g., GA) have higher BC values and those in some states in the southwest and mid-west (e.g., AZ and MO) have lower BC values. Networks of wells in some other states (e.g., PA and SC) have a good mix of high and low BC values.

#### 4.2.3. Closeness Centrality Results

**Figure 10:**
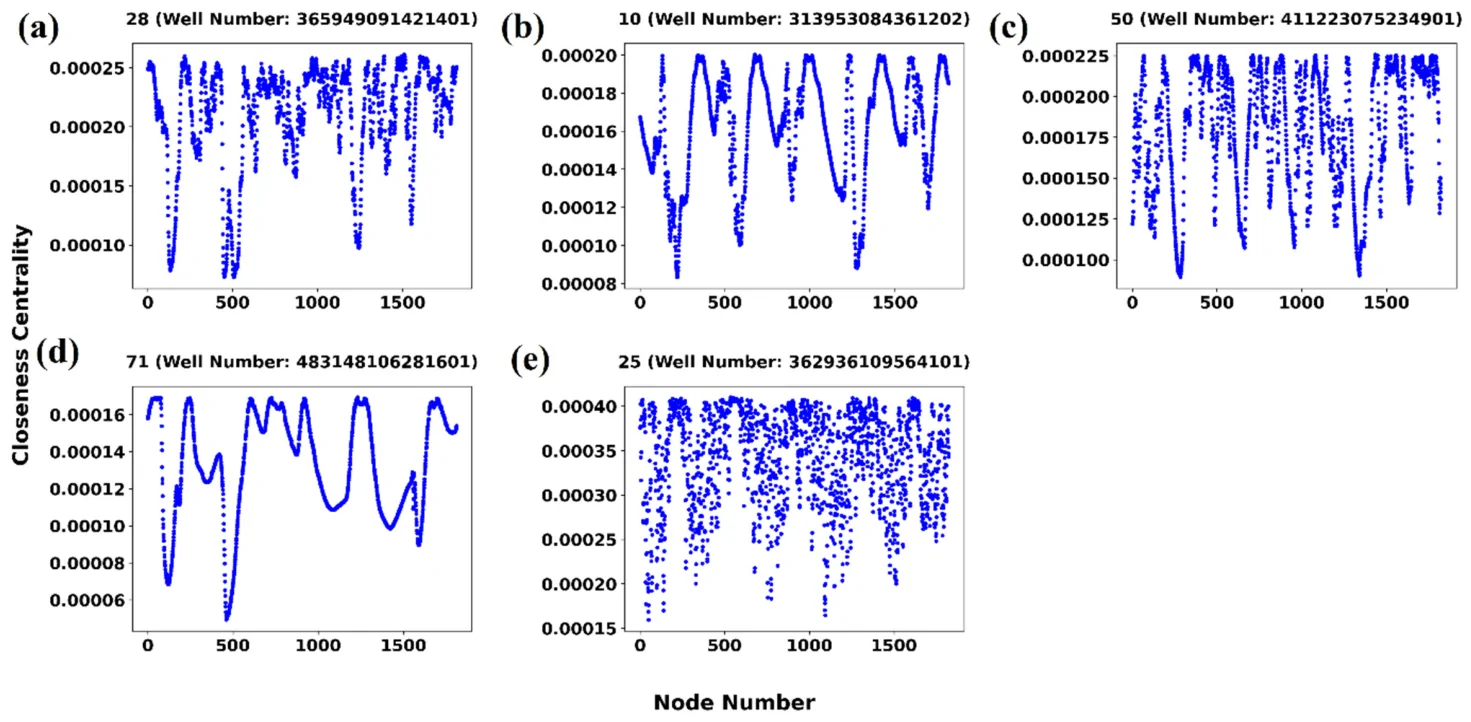
Closeness centrality values for groundwater-level networks from five selected wells: (**a**) Well 28 (Howell, MO); (**b**) Well 10 (Randolph, GA); (**c**) Well 50 (Monroe, PA); (**d**) Well 71 (Valley, MT); and (**e**) Well 25 (Apache, AZ).

Figure 10a–e shows the closeness centrality (CC) values obtained for the different nodes in the groundwater-level networks from Wells 28, 10, 50, 71, and 25, respectively. The ranges of the CC values of nodes for the five networks are Well 28: 0.000073–0.000261; Well 10: 0.000083–0.00020; Well 50: 0.000089–0.000226; Well 71: 0.000049–0.000169; and Well 25: 0.000159–0.00041. The mean CC values for these five networks are 0.000208, 0.000163, 0.00018, 0.000134, and 0.000341, respectively. The mean CC values suggest that the nodes in the groundwater-level network from Well 25 (Apache, AZ) (Figure 10e (CC = 0.000341) are the closest among themselves when compared with the nodes in the other networks, suggesting that the nodes in the former network can spread the information much more quickly within the network. Most of the nodes in this network have CC values above 0.00025, while the values of the nodes in the other four networks are generally below 0.00025, with the nodes in the network for Well 71 (Valley, MT) having far lower values, i.e., below 0.00017. The nodes in each of the networks from Well 28 (Howell, MO) (Figure 10a), Well 10 (Randolph, GA) (Figure 10b), and Well 50 (Monroe, PA) (Figure 10c) have somewhat similar levels of closeness among themselves, with only minor differences. The nodes in the network from Well 71 (Valley, MT) (Figure 10d) are the farthest among themselves, suggesting that the spread of information is much slower in the network. The nodes in the network from Well 25 (Apache, AZ) have a significantly more even distribution of CC values, followed by that in Well 50 (Monroe, PA) and Well 28 (Howell, MO). The nodes in the networks from Well 10 (Randolph, GA) and Well 71 (Valley, MT) have significant variability in CC values.

**Figure 11:**
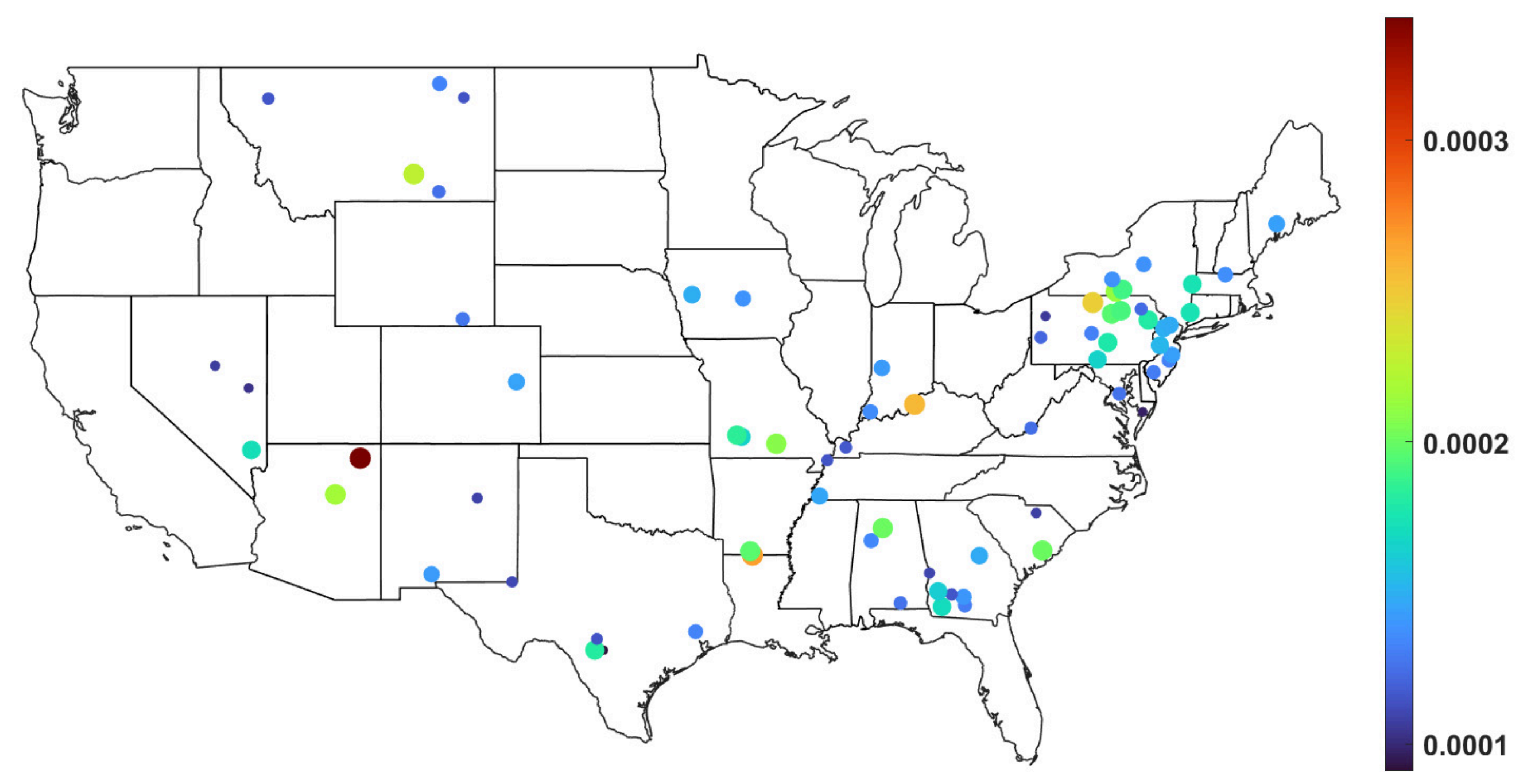
Closeness centrality results for groundwater-level networks from all of the 71 wells for the optimal distance thresholds.

Figure 11 presents the CC values obtained for the 71 groundwater-level networks. Considering all the 71 networks, the CC values are in the range 0.00009–0.00034 (Table 2). The highest CC value of 0.00034 is obtained for the network from Well 25 (Apache, AZ), followed by a CC value of 0.00027 for the network from Well 14 (Claiborne Parish, LA), 0.00026 for the network from Well 34 (Clark, IN), and 0.00025 for the network from Well 57 (Potter, PA). The lowest CC value of 0.00009 is obtained for the network from Well 1 (Medina, TX), followed by a CC value of 0.0001 for the networks from two wells: Well 32 (Somerset, MD) and Well 36 (White Pine, NV). Eight wells have CC ≥ 0.20, while over 45 wells have CC ≤ 0.15. The CC values of the groundwater-level networks do not exhibit any region-specific patterns. However, the networks in some states (e.g., AZ, AR, and LA, and, to a certain extent, MO) have relatively higher CC values, while the networks in some other states (e.g., IA and NM) have relatively lower CC values. Networks of groundwater levels in some other states (e.g., AL, IN, MT, and PA) have a good mix of high and low CC values.

#### 4.2.4. Clustering Coefficient Results

**Figure 12:**
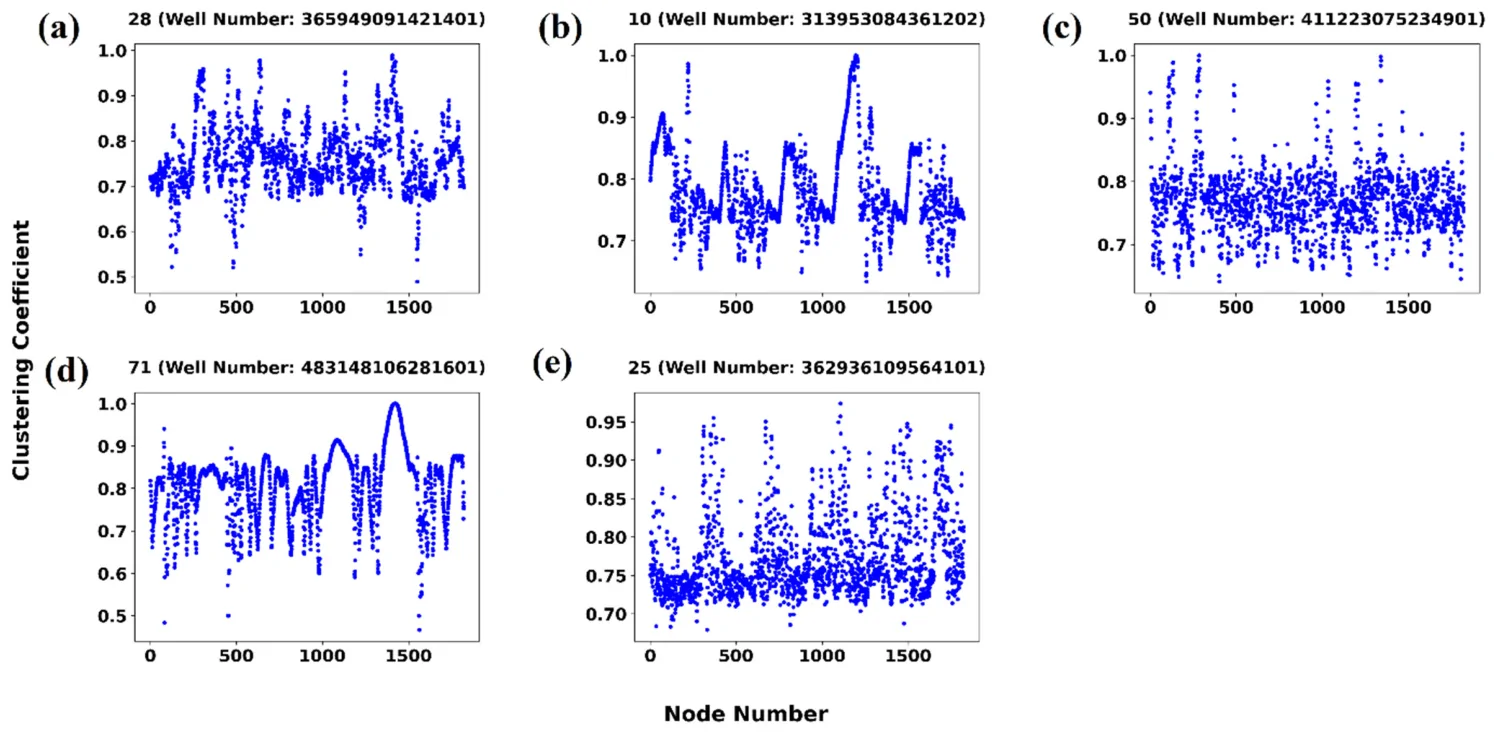
Clustering coefficient values for groundwater-level networks from five selected wells: (**a**) Well 28 (Howell, MO); (**b**) Well 10 (Randolph, GA); (**c**) Well 50 (Monroe, PA); (**d**) Well 71 (Valley, MT); and (**e**) Well 25 (Apache, AZ).

Figure 12a–e shows the clustering coefficient (C_c_) values obtained for the different nodes in each of the five selected groundwater-level networks, i.e., Wells 28, 10, 50, 71, and 25. The C_c_ values indicate different connectivity levels for different nodes in the networks. The range of C_c_ values for the network from Well 28 is 0.489–0.989, Well 10 is 0.634–1.0, Well 50 is 0.641–1.0, Well 71 is 0.467–1.0, and Well 25 is 0.679–0.974. The mean C_c_ values for these five networks are 0.760, 0.781, 0.770, 0.809, and 0.772, respectively (Table 2). These generally high C_c_ values (>0.75) suggest that each of these five groundwater networks has significant local connectivity (clustering) and there is no significant difference in the extent of clustering within these networks, with the exception of the network from Well 71 (Valley, MT), which has a noticeably higher value (0.809) when compared with the others (0.760–0.781).

In each of these five networks, most of the nodes have C_c_ > 0.70, which indicates a significant amount of clustering. Indeed, almost all of the nodes in the groundwater-level network of Well 25 (Apache, AZ) have C_c_ > 0.70 (Figure 12e). However, only a small number of nodes have very high local connectivity, with C_c_ > 0.90. Several nodes in the groundwater-level network of Well 28 (Howell, MO) (Figure 12a) and of Well 71 (Valley, MT) (Figure 12d) have C_c_ < 0.60. Further, a significant majority of nodes in each of these five groundwater-level networks have C_c_ values in the range 0.70–0.80, and this is particularly the case for Well 50 (Monroe, PA) (Figure 12c). There is a slightly more even distribution of C_c_ values for Well 25 (Apache, AZ) (Figure 12e), and the network of Well 10 (Randolph, GA) seems to have significant variations in the distribution of C_c_ values (Figure 12b).

**Figure 13:**
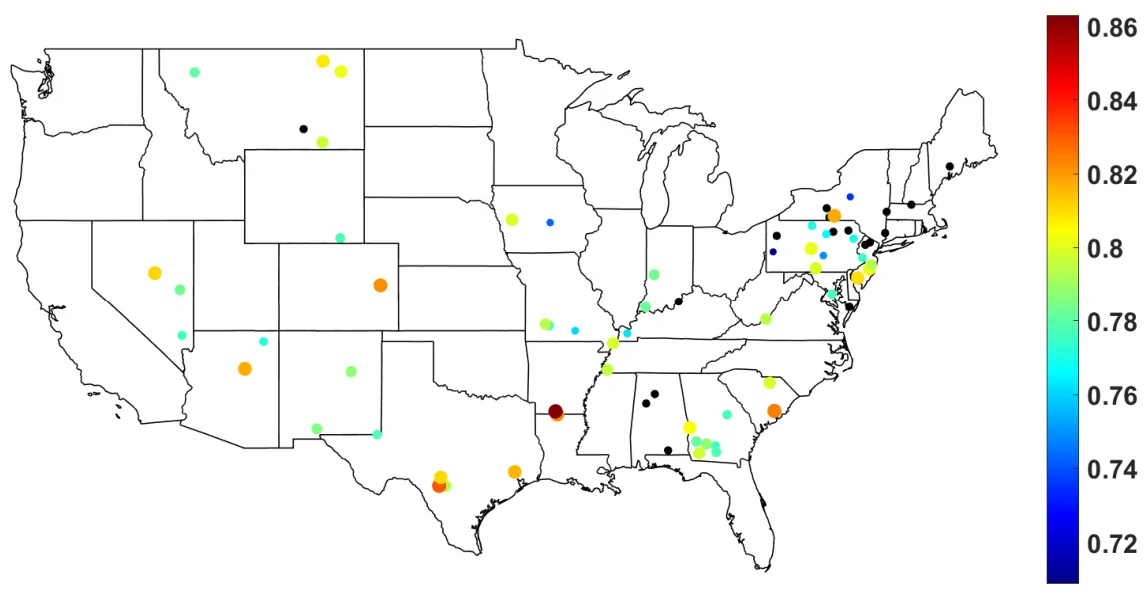
Clustering coefficient results for groundwater-level networks from all of the 71 wells for the optimal distance thresholds.

Figure 13 presents the clustering coefficient values obtained for the 71 groundwater-level networks; see also Table 2. The clustering coefficient values are in the range 0.709–0.863. The highest C_c_ value of 0.863 is obtained for the network from Well 15 (Union, AR), followed by a value of 0.858 for the network from Well 34 (Clark, IN). The lowest C_c_ value of 0.709 is obtained for the network from Well 46 (Allegheny, PA), followed by 0.717 for the network from Well 55 (Sullivan, PA). Among all of the 71 groundwater-level networks, more than a quarter of the networks (18) have C_c_ values of greater than 0.800. There do not seem to be any regional patterns in the groundwater-level networks for local clustering. However, the groundwater-level networks in some southeastern states (e.g., AL and GA) have relatively lower C_c_ values (lower clustering). On the other hand, networks in some south/southwestern states (e.g., NV, AZ, TX, CO, LA, and AR, and, to some extent, NM) and southeastern states (e.g., SC) have relatively higher C_c_ values (higher clustering). Networks of groundwater levels in some northwestern (e.g., MT) and eastern (e.g., PA, NY, and NJ) states have high as well as low C_c_ values.

To examine whether the network measures capture nonlinear patterns in the groundwater level data, a surrogate analysis is performed for data from Well 28 (which shows significantly different results in terms of phase space and FNN analysis; see Figure 3 and Figure 4) using 100 surrogate series. The same phase-space reconstruction and network-construction procedures are applied independently to each surrogate series. The complete methodology and results are provided in Section S1, Table S1, and Figure S1 of the Supplementary Materials.

### 4.3. Relationships Between Groundwater-Level Statistics and Network Measures

**Figure 14:**
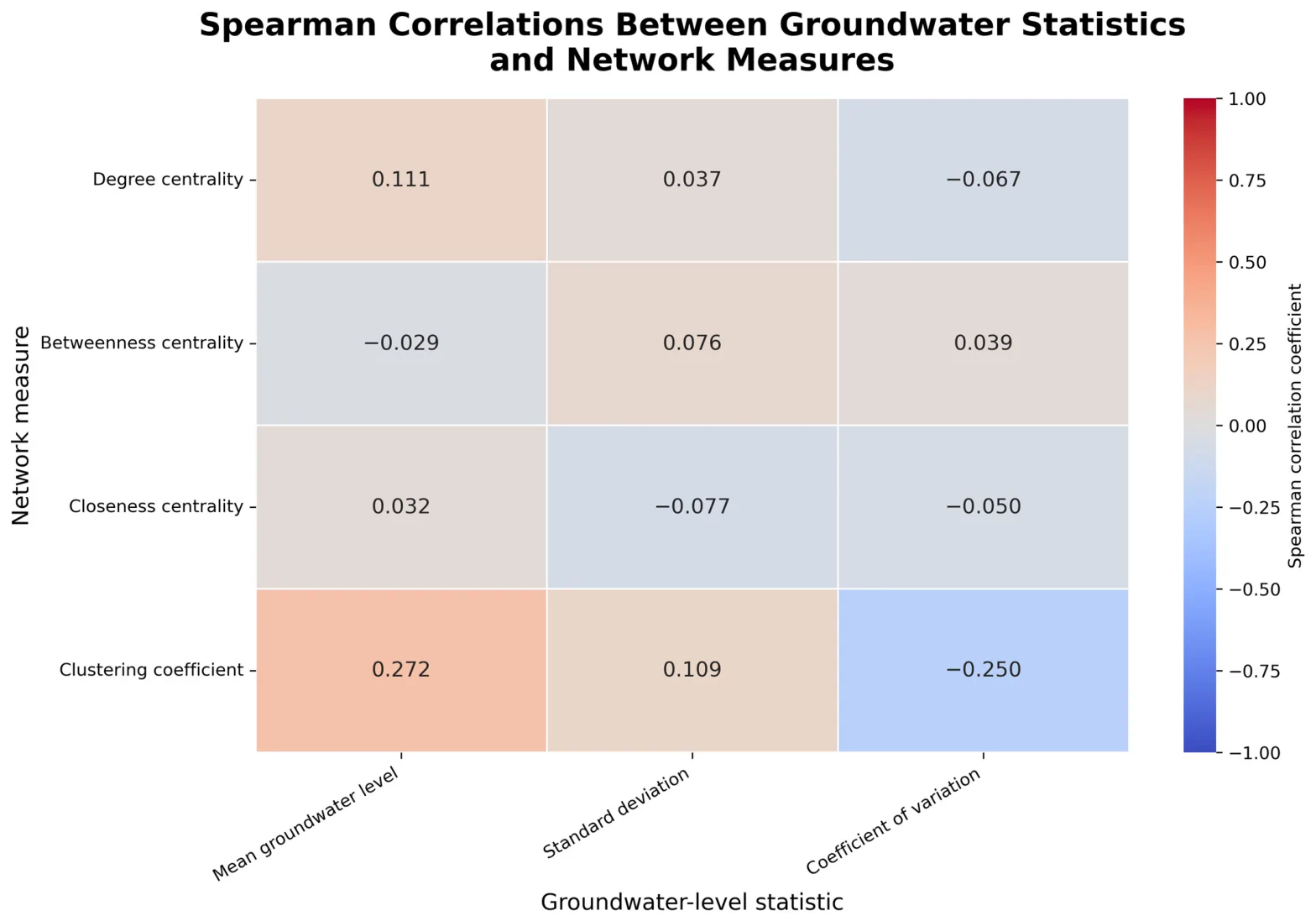
Spearman correlation coefficients between three groundwater-level statistics (mean, standard deviation, and coefficient of variation) and four network measures (degree centrality, betweenness centrality, closeness centrality, and clustering coefficient) for the 71 wells. None of the correlations is statistically significant after applying the Benjamini–Hochberg false-discovery-rate correction at the 0.05 significance level.

Figure 14 presents the Spearman correlation coefficients between the three groundwater-level statistics (mean, standard deviation, and coefficient of variation) and the four network measures (degree centrality, betweenness centrality, closeness centrality, and clustering coefficient) for the 71 wells. Degree centrality shows weak correlations with the mean groundwater level (*ρ* = 0.111), standard deviation (*ρ* = 0.037), and coefficient of variation (*ρ* = −0.067). Betweenness centrality also shows weak correlations with the mean groundwater level (*ρ* = −0.029), standard deviation (*ρ* = 0.076), and coefficient of variation (*ρ* = 0.039). Similarly, closeness centrality shows weak correlations with the mean groundwater level (*ρ* = 0.032), standard deviation (*ρ* = −0.077), and coefficient of variation (*ρ* = −0.050).

The clustering coefficient shows weak correlations with all three groundwater-level statistics: a positive correlation with the mean groundwater level (*ρ* = 0.272), a positive correlation with the standard deviation (*ρ* = 0.109), and a negative correlation with the coefficient of variation (*ρ* = −0.250). Although the correlations with the mean groundwater level (raw *p* = 0.0216) and coefficient of variation (raw *p* = 0.0359) are statistically significant before correction, neither remains significant after the false-discovery-rate correction (adjusted *p* = 0.215). The correlation with the standard deviation is also statistically non-significant (raw *p* = 0.367, adjusted *p* = 0.813).

Overall, none of the 12 correlations is statistically significant after correction for multiple comparisons. Thus, the network properties are not systematically associated with the mean, standard deviation, or coefficient of variation of the groundwater levels. This result suggests that the variations in the network measures are not explained by these basic statistical characteristics alone.

## 5. Conclusions

The present study applies complex network theory to examine the temporal dynamics of groundwater levels. This is the first ever study to combine chaos theory and complex network theory to examine groundwater-level dynamics. This approach helps quantify and compare the complexity and temporal dynamics and connectivity in groundwater-levels in a region. Daily groundwater-level data observed over the period 2010–2014 from 71 wells across the United States are considered. Each well is considered as a network. For each well, the groundwater-level time series is reconstructed in a multidimensional phase space, and the false nearest neighbour (FNN) method is used to determine the optimal embedding dimension. The reconstructed time series is then converted into a network. The properties of the 71 groundwater-level networks are analysed using four complex networks-based measures, namely degree centrality, betweenness centrality, closeness centrality, and clustering coefficient. In addition, Spearman rank correlation analysis is performed to determine whether variations in these network measures are related to the mean, standard deviation, and coefficient of variation of the groundwater-level records.

The optimal embedding dimensions from the FNN method for phase-space reconstruction and subsequent network construction of the 71 groundwater level time series are found to range from 4 (for nine wells) to 18 (for one well). These dimensions suggest a wide range of complexity in the 71 groundwater level time series. For a large majority (i.e., 62) of the 71 series, optimal embedding dimensions are found to be in the range of 4–10, suggesting a low-to-medium-level complexity of the groundwater level dynamics. Optimal embedding dimensions greater than 10 are observed only for nine of the 71 groundwater level series, which may be considered to indicate higher level of complexity.

The degree centrality values for the 71 groundwater-level networks are in the range 0.12–0.55. The betweenness centrality values are in the range 604–4750. The closeness centrality values are in the range 0.00009–0.00034. The clustering coefficient values are in the range 0.709–0.863. The results are generally consistent for the four network measures across the 71 groundwater-level networks. The network measures do not seem to exhibit any region-specific patterns. However, depending upon the measure, networks of wells in some states have higher/lower values and networks in some other states have a good mix of high and low values. For instance, groundwater levels in some southwestern states exhibit better temporal connectivity when compared with those in some southeastern states, while groundwater levels in some northwestern and eastern states have a mix of high and low connectivity. The network measures also seem to suggest the role of seasonality on network connectivity.

Spearman rank correlation analysis shows no statistically significant relationships between the mean, standard deviation, and coefficient of variation of the groundwater levels and any of the four network measures after adjusting the *p*-values for the 12 correlations examined. This result indicates that the differences in network properties among the wells are not explained by these basic statistical characteristics alone.

The results from the present study are encouraging regarding the usefulness of the complex network-based concepts for examining the temporal connectivity in groundwater levels. However, the study also has certain limitations. First, the FNN method is used in this study to determine the optimal embedding dimension for phase space reconstruction for identification of nodes for network construction. While the FNN method often yields reliable results, including for short and noisy data, the exact identification of the optimal dimension (i.e., the dimension corresponding to the lowest %FNN value) is sometimes not easy. Second, optimum or critical distance threshold (*DT_c_*) for the identification of links between a pair of nodes is determined by calculating the network density for different distance thresholds and determining the threshold at which the slope of network density is maximum. While this approach is found to perform well for many different types of data in hydrology (including for the groundwater level time series in the present study), there can also be situations where the approach may not perform well. Third, the present study focuses only on the temporal behaviour of groundwater levels and does not consider other factors that may influence groundwater dynamics. These factors include aquifer characteristics, rainfall, recharge, groundwater extraction, land use, and pumping activities. Future studies should consider these factors along with the complex network measures to better explain the differences observed among the wells and regions. Fourth, the four network measures applied in this study are some of the basic measures and, therefore, can reveal only certain aspects of the temporal connectivity of groundwater levels. There are several other measures that reveal additional properties of network connectivity, such as shortest path length, assortativity, and community detection, among others. More recent optimal-transport-based approaches, such as the fused unbalanced Gromov–Wasserstein framework [65], may further complement these measures by enabling comparisons of groundwater recurrence networks across wells, aquifer settings, and hydrological conditions and by supporting the assessment of their responses to disturbances. Addressing these issues, and other relevant ones, is important for improving our understanding of the temporal connectivity and dynamics of groundwater levels.

## Figures and Tables

**Table 1:** Some basic details of the 71 groundwater wells considered in this study and statistics of groundwater levels.

Sl. No.	Well Number	Lat	Lon	Location County, State	Mean (ft/Day)	Std. Dev. (ft/Day)	CV
1	292618099165901	29.44	99.28	Medina, TX	202.45	35.25	0.17
2	292628099401401	29.45	99.66	Uvalde, TX	66.65	5.16	0.08
3	295204099340201	29.87	99.57	Bandera, TX	249.52	1.60	0.01
4	300849095143301	30.15	95.24	Montgomery, TX	140.04	9.35	0.07
5	310507084262201	31.09	84.44	Mitchell, GA	53.04	4.50	0.08
6	310813083260301	31.14	83.43	Cook, GA	181.23	2.14	0.01
7	311319086153601	31.22	86.26	Covington, AL	10.35	3.35	0.32
8	312712082593301	31.45	83.49	Tift, GA	140.62	4.55	0.03
9	313247084005001	31.55	84.01	Dougherty, GA	52.31	4.16	0.08
10	313953084361202	31.66	84.60	Randolph, GA	192.90	24.75	0.13
11	321745106492106	32.30	106.82	Dona Ana, NM	39.22	3.21	0.08
12	322036084590301	32.34	84.99	Chattahooche, GA	50.43	1.31	0.03
13	325848082480901	32.98	82.80	Washington, GA	250.57	3.41	0.01
14	330002092445901	33.00	92.75	Claiborne Parish, LA	252.54	3.56	0.01
15	330855092505601	33.15	92.85	Union, AR	297.06	4.78	0.02
16	331022080021801	33.17	80.04	Berkeley, SC	67.44	0.81	0.01
17	333204087324601	33.53	87.55	Tuscaloosa, AL	40.48	2.54	0.06
18	335929087021001	33.99	87.04	Cullman, AL	39.44	4.01	0.10
19	343201080182001	34.53	80.31	Chesterfield, SC	104.35	2.64	0.03
20	350414104485101	35.07	104.81	Guadalupe, NM	366.86	4.10	0.01
21	350900089482300	35.15	89.81	Shelby, TN	104.06	0.96	0.01
22	351214111022101	35.20	111.04	Coconino, AZ	329.56	0.14	0.00
23	362500089281101	36.42	89.47	Lake, TN	18.46	2.59	0.14
24	362714103071201	32.01	103.29	Lea, NM	175.86	2.97	0.02
25	362936109564101	36.49	109.95	Apache, AZ	374.42	0.13	0.00
26	364650114432001	36.78	114.72	Clark, NV	396.00	0.41	0.00
27	365210088391301	36.87	88.65	Graves, KY	7.30	1.32	0.18
28	365949091421401	37.00	91.70	Howell, MO	320.00	21.88	0.07
29	371435093134701	37.24	93.23	Greene, MO	352.35	7.28	0.02
30	371801093260401	37.30	93.43	Greene, MO	184.52	7.40	0.04
31	373234080320101	37.54	80.53	Monroe, WV	84.20	3.96	0.05
32	380616075380701	38.11	75.64	Somerset, MD	3.04	1.83	0.60
33	380626087344401	38.11	87.58	Vanderburgh, IN	21.98	1.24	0.06
34	382156085382401	38.37	85.64	Clark, IN	33.24	3.01	0.09
35	384333076394701	38.73	76.66	Calvert, MD	175.10	2.66	0.02
36	385521114503601	38.92	114.84	White Pine, NV	431.90	2.86	0.01
37	390740103055801	39.13	103.10	Kit Carson, CO	48.68	1.13	0.02
38	392733075092401	39.46	75.16	Cumberland, NJ	174.88	1.09	0.01
39	393619087043001	39.61	87.08	Parke, IN	14.38	1.00	0.07
40	394036116183401	39.68	116.31	Eureka, NV	38.72	0.24	0.01
41	395150074284201	39.86	74.48	Burlington, NJ	22.57	1.85	0.08
42	395322077365301	39.89	77.61	Franklin, PA	73.44	7.22	0.10
43	400232074213201	40.04	74.36	Ocean, NJ	26.80	0.90	0.03
44	402151074525301	40.36	74.88	Hunterdon, NJ	2.62	1.77	0.68
45	402735077100901	40.46	77.17	Perry, PA	12.56	1.72	0.14
46	403734080063001	40.63	80.11	Allegheny, PA	6.42	0.69	0.11
47	404556077525101	40.77	77.88	Centre, PA	88.49	20.28	0.23
48	405613074430901	40.94	74.72	Sussex, NJ	13.07	1.29	0.10
49	410207074270001	41.04	74.45	Morris, NJ	4.10	0.70	0.17
50	411223075234901	41.21	75.40	Monroe, PA	11.02	1.57	0.14
51	411412105272001	41.24	105.46	Albany, WY	33.62	8.41	0.25
52	411958079540202	41.33	79.90	Venango, PA	108.91	6.74	0.06
53	412427076594401	41.41	76.99	Lycoming, PA	99.56	1.67	0.02
54	412714073331301	41.45	73.55	Putnam, NY	2.71	0.93	0.34
55	413026076352901	41.51	76.59	Sullivan, PA	26.51	2.00	0.08
56	413346075421301	41.56	75.70	Lackawanna, PA	54.82	2.26	0.04
57	414640077493801	41.78	77.83	Potter, PA	21.17	5.88	0.28
58	415439093094801	41.91	93.16	Marshall, IA	13.32	2.40	0.18
59	420212095235701	42.04	95.40	Crawford, IA	26.71	1.74	0.07
60	420828076484601	42.14	76.81	Chemung, NY	23.24	0.71	0.03
61	421213076313301	42.20	76.53	Tioga, NY	18.18	3.02	0.17
62	422241073274601	42.38	73.46	Columbia, NY	10.25	0.93	0.09
63	423143076582601	42.53	76.97	Yates, NY	4.41	0.93	0.21
64	424057072010301	42.68	72.02	Worcester, MA	25.81	0.87	0.03
65	430056075354102	43.02	75.59	Madison, NY	7.52	1.52	0.20
66	441801069455501	44.30	69.77	Kennebec, ME	11.69	1.70	0.15
67	451746106301101	45.30	106.50	Rosebud, MT	9.30	0.92	0.10
68	455016107360402	45.84	107.60	Big Horn, MT	5.86	0.98	0.17
69	480550113585001	48.10	113.98	Flathead, MT	5.83	2.25	0.39
70	480728105243901	48.12	105.41	Roosevelt, MT	13.96	1.70	0.12
71	483148106281601	48.53	106.47	Valley, MT	6.83	0.84	0.12

**Table 2:** Results from network analysis of groundwater levels in the 71 wells across the United States: *m_opt_*—optimal embedding dimension obtained from the FNN algorithm; *DT_c_*—critical distance threshold; DC—degree centrality; BC—betweenness centrality; CC—closeness centrality; C_c_–clustering coefficient.

Sl. No.	Well Number	m_opt_	DT_c_	DC	BC	CC	C_c_
1	292618099165901	6	0.400	0.136	4750	0.00009	0.794
2	292628099401401	10	0.659	0.427	2401	0.00018	0.830
3	295204099340201	5	0.328	0.173	3691	0.00011	0.811
4	300849095143301	4	0.266	0.189	3159	0.00013	0.816
5	310507084262201	5	0.498	0.231	2190	0.00017	0.798
6	310813083260301	6	0.396	0.161	3124	0.00013	0.779
7	311319086153601	6	0.390	0.148	3243	0.00013	0.757
8	312712082593301	4	0.261	0.183	2923	0.00014	0.775
9	313247084005001	5	0.331	0.156	3627	0.00012	0.788
10	313953084361202	10	0.932	0.221	2286	0.00016	0.781
11	321745106492106	5	0.327	0.186	2879	0.00014	0.786
12	322036084590301	5	0.330	0.144	3886	0.00011	0.804
13	325848082480901	5	0.328	0.193	2730	0.00015	0.776
14	330002092445901	5	1.160	0.377	1009	0.00027	0.822
15	330855092505601	5	0.827	0.314	1695	0.00020	0.863
16	331022080021801	5	0.329	0.312	1882	0.00020	0.824
17	333204087324601	7	0.438	0.171	3057	0.00014	0.723
18	335929087021001	4	0.233	0.284	1715	0.00020	0.777
19	343201080182001	6	0.392	0.151	3974	0.00011	0.799
20	350414104485101	6	0.395	0.141	3894	0.00011	0.788
21	350900089482300	5	0.330	0.226	2839	0.00015	0.796
22	351214111022101	18	2.001	0.270	1522	0.00022	0.818
23	362500089281101	7	0.465	0.153	3657	0.00012	0.799
24	362714103071201	6	0.396	0.147	3831	0.00011	0.778
25	362936109564101	4	0.593	0.465	604	0.00034	0.772
26	364650114432001	6	0.531	0.213	2147	0.00017	0.776
27	365210088391301	5	0.322	0.143	3580	0.00012	0.762
28	365949091421401	10	0.855	0.292	1701	0.00021	0.760
29	371435093134701	6	0.397	0.215	2360	0.00017	0.778
30	371801093260401	4	0.394	0.243	1957	0.00018	0.794
31	373234080320101	14	0.770	0.262	3607	0.00012	0.794
32	380616075380701	9	0.563	0.145	4322	0.00010	0.782
33	380626087344401	7	0.439	0.166	2991	0.00014	0.782
34	382156085382401	5	0.310	0.548	1264	0.00026	0.858
35	384333076394701	4	0.264	0.165	3195	0.00013	0.777
36	385521114503601	5	0.333	0.146	4070	0.00010	0.782
37	390740103055801	12	0.795	0.286	3108	0.00015	0.821
38	392733075092401	6	0.383	0.182	3268	0.00013	0.810
39	393619087043001	6	0.393	0.182	2835	0.00014	0.783
40	394036116183401	11	0.672	0.159	4035	0.00011	0.811
41	395150074284201	8	0.530	0.173	3176	0.00013	0.803
42	395322077365301	9	0.589	0.311	2634	0.00017	0.799
43	400232074213201	10	0.654	0.204	2944	0.00014	0.793
44	402151074525301	6	0.386	0.223	2642	0.00015	0.775
45	402735077100901	6	0.547	0.206	2004	0.00018	0.750
46	403734080063001	7	0.446	0.121	3403	0.00012	0.709
47	404556077525101	4	0.266	0.214	3289	0.00013	0.802
48	405613074430901	8	0.444	0.173	2687	0.00015	0.736
49	410207074270001	9	0.549	0.159	2708	0.00015	0.735
50	411223075234901	6	0.567	0.223	2022	0.00018	0.770
51	411412105272001	5	0.333	0.191	3318	0.00013	0.778
52	411958079540202	4	0.266	0.140	3967	0.00011	0.772
53	412427076594401	6	0.558	0.247	1778	0.00020	0.766
54	412714073331301	6	0.361	0.376	2313	0.00018	0.821
55	413026076352901	7	0.386	0.213	1669	0.00019	0.717
56	413346075421301	7	0.423	0.157	3330	0.00012	0.788
57	414640077493801	8	1.195	0.304	1206	0.00025	0.770
58	415439093094801	12	0.729	0.161	2866	0.00014	0.742
59	420212095235701	4	0.263	0.212	2692	0.00015	0.799
60	420828076484601	8	0.587	0.257	1499	0.00021	0.759
61	421213076313301	7	0.461	0.357	2112	0.00019	0.817
62	422241073274601	10	0.632	0.253	2280	0.00018	0.753
63	423143076582601	12	0.663	0.189	2932	0.00014	0.760
64	424057072010301	5	0.320	0.180	2914	0.00014	0.756
65	430056075354102	16	1.355	0.154	2795	0.00014	0.734
66	441801069455501	5	0.329	0.239	2874	0.00014	0.784
67	451746106301101	15	0.960	0.174	3403	0.00013	0.797
68	455016107360402	8	0.499	0.317	1445	0.00023	0.781
69	480550113585001	7	0.457	0.186	3692	0.00012	0.781
70	480728105243901	5	0.333	0.146	3703	0.00011	0.802
71	483148106281601	16	1.048	0.174	3072	0.00013	0.809

## Data Availability

We thank the U.S. Geological Survey (USGS) for making the groundwater data publicly available. The USGS groundwater data can be obtained from the National Water Dashboard (NWD) at: https://dashboard.waterdata.usgs.gov/app/nwd/en/ (accessed on 10 March 2026).

## Supplementary Materials

The following supporting information can be downloaded at: https://www.mdpi.com/article/10.3390/e28101068/s1, Figure S1: Comparison of the observed and surrogate network measures for Well 28 (Howell, Missouri); (a) degree centrality, (b) betweenness centrality, (c) closeness centrality, and (d) clustering coefficient. The distributions are obtained from 100 surrogate groundwater-level series. The red solid lines indicate the observed values, and the black dashed lines indicate the surrogate means; Table S1: Comparison of the observed network measures for Well 28 (Howell, Missouri) with the distributions obtained from 100 surrogate groundwater-level series.
