# Supplementary material for: Temporal Dynamics of Groundwater Levels: A Complex Networks-Based Approach
Source: Entropy (Basel). 2026 Sep 28;28(10):1068. doi: 10.3390/e28101068 (PMC13703553; doi:10.3390/e28101068)
Supplement: Supplementary file 1 [file entropy-28-01068-s001.zip › entropy-4530531-supplementary.pdf]

entropy

MDPI

Temporal Dynamics of Groundwater Levels: A Complex Networks-Based Approach

Bhadran Deepthi [1] and Bellie Sivakumar [2,*]

[1] Kerala State Council for Science, Technology and Environment, Pattom, Thiruvananthapuram 695004, India; deepthibhadran2@gmail.com
[2] Department of Civil Engineering, Indian Institute of Technology Bombay, Powai, Mumbai 400076, India
* Correspondence: b.sivakumar@iitb.ac.in

Supplementary Material

S1. Surrogate Analysis

A surrogate analysis is conducted for Well 28 (Howell, Missouri) (which shows significant differences in terms of phase space and false nearest neighbor results when compared to the other four wells considered for illustration) to examine whether the observed network measures may arise from the phase-space reconstruction and network-construction procedures alone. A total of 100 surrogate groundwater-level series are generated from the observed record. Each surrogate series is analyzed independently using the same procedure applied to the observed series. The optimal embedding dimension is determined using the false nearest neighbor (FNN) method, and the critical distance threshold is determined independently for each surrogate using the network-density method. The resulting networks are evaluated using degree centrality, betweenness centrality, closeness centrality, and clustering coefficient.

For each network measure, the observed value is compared with the distribution obtained from the 100 surrogate networks. The surrogate mean, standard deviation, and 95% percentile interval are calculated. A Z-score is computed as the difference between the observed value and the surrogate mean divided by the surrogate standard deviation. Two-sided empirical p-values are calculated, and the four p-values are adjusted using the Benjamini–Hochberg false-discovery-rate procedure [1]. A measure is considered statistically significant when its adjusted p-value is less than 0.05.

S2. Results

The observed and surrogate network measures for Well 28 are compared in Table S1, while their distributions are presented in Figure S1. The observed degree centrality is significantly lower than the surrogate mean, whereas the observed betweenness centrality is significantly higher. The observed closeness centrality is also significantly lower than the surrogate mean. These three measures remain sig nificant after the false-discovery-rate correction. In contrast, the observed clustering coefficient lies within the 95% surrogate interval and does not differ significantly from the surrogate distribution.

 https://doi.org/10.3390/e28101068

The results indicate that the network-construction procedure applied to the surro gate series does not reproduce all the connectivity characteristics of the observed network from Well 28. However, the clustering behavior of the observed network is comparable to that of the surrogate networks. Because this analysis is carried out for only one well, the findings are specific to Well 28 and are not generalized to all 71 wells.

Table S1. Comparison of the observed network measures for Well 28 (Howell, Missouri) with the distributions obtained from 100 surrogate groundwater-level series.

**Network measure**	**Observed**	**Surrogate mean**	**Surrogate SD**	**95% surrogate interval**	**Z-score**	**FDR-adjusted p-value**
Degree centrality	0.292	0.5005	0.0328	0.4429–0.5604	−6.365	0.013
Betweenness centrality	1701	457.598	31.492	401.698–513.899	39.483	0.013
Closeness centrality	0.000210	0.000374	0.00000881	0.000359–0.000390	−18.632	0.013
Clustering coefficient	0.760	0.7400	0.0167	0.7121–0.7691	1.201	0.277

https://doi.org/10.3390/e28101068

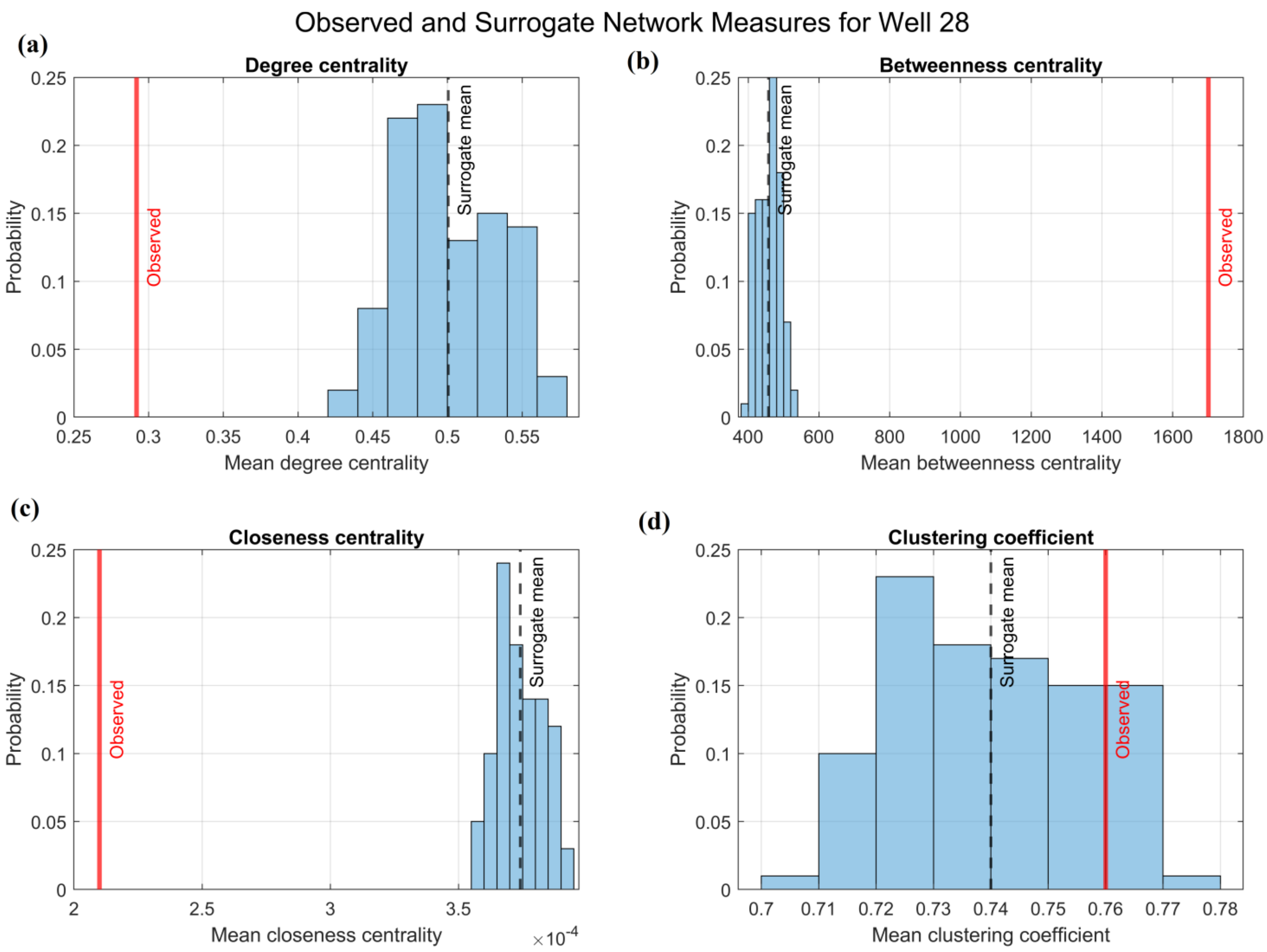

Figure S1. Comparison of the observed and surrogate network measures for Well 28 (Howell, Missouri): (a) degree centrality, (b) betweenness centrality, (c) closeness centrality, and (d) clustering coefficient. The distributions are obtained from 100 surrogate groundwater-level series. The red solid lines indicate the observed values, and the black dashed lines indicate the surrogate means.

Reference

1. Benjamini, Y.; Hochberg, Y. Controlling the false discovery rate: A practical and powerful approach to multiple testing. *Journal of the Royal Statistical Society: Series B (Methodological)* **1995**, *57*, 289–300. https://doi.org/10.1111/j.2517-6161.1995.tb02031.x.
